# *Corynebacterium jeikeium jk0268* Constitutes for the 40 Amino Acid Long PorACj, Which Forms a Homooligomeric and Anion-Selective Cell Wall Channel

**DOI:** 10.1371/journal.pone.0075651

**Published:** 2013-10-08

**Authors:** Narges Abdali, Enrico Barth, Amir Norouzy, Robert Schulz, Werner M. Nau, Ulrich Kleinekathöfer, Andreas Tauch, Roland Benz

**Affiliations:** 1 School of Engineering and Science, Jacobs University Bremen, Bremen, Germany; 2 Rudolf Virchow Center, DFG-Research Center for Experimental Biomedicine, University of Würzburg, Würzburg, Germany; 3 Institute for Genome Research and Systems Biology Center for Biotechnology (CeBiTec), Bielefeld University, Bielefeld, Germany; Centre National de la Recherche Scientifique, Aix-Marseille Université, France

## Abstract

*Corynebacterium jeikeium*, a resident of human skin, is often associated with multidrug resistant nosocomial infections in immunodepressed patients. *C. jeikeium* K411 belongs to mycolic acid-containing actinomycetes, the mycolata and contains a channel-forming protein as judged from reconstitution experiments with artificial lipid bilayer experiments. The channel-forming protein was present in detergent treated cell walls and in extracts of whole cells using organic solvents. A gene coding for a 40 amino acid long polypeptide possibly responsible for the pore-forming activity was identified in the known genome of *C. jeikeium* by its similar chromosomal localization to known *porH* and *porA* genes of other *Corynebacterium* strains. The gene *jk0268* was expressed in a porin deficient *Corynebacterium glutamicum* strain. For purification temporarily histidine-tailed or with a GST-tag at the N-terminus, the homogeneous protein caused channel-forming activity with an average conductance of 1.25 nS in 1M KCl identical to the channels formed by the detergent extracts. Zero-current membrane potential measurements of the voltage dependent channel implied selectivity for anions. This preference is according to single-channel analysis caused by some excess of cationic charges located in the channel lumen formed by oligomeric alpha-helical wheels. The channel has a suggested diameter of 1.4 nm as judged from the permeability of different sized hydrated anions using the Renkin correction factor. Surprisingly, the genome of *C. jeikeium* contained only one gene coding for a cell wall channel of the PorA/PorH type found in other *Corynebacterium* species. The possible evolutionary relationship between the heterooligomeric channels formed by certain *Corynebacterium* strains and the homooligomeric pore of *C. jeikeium* is discussed.

## Introduction

Members of the genus *Corynebacterium* are of considerable interest because some are potent producers of glutamate, lysine and other amino acids through fermentation processes on an industrial scale. Prominent examples of amino acid producers are *Corynebacterium glutamicum* or *Corynebacterium callunae* [1]–[6]. These bacteria belong to the family Corynebacteriaceae that is a distinctive suprageneric actinomycete taxon, the mycolata, which also includes mycobacteria, nocardiae, rhodococci and closely related genera. The mycolata share with the genus *Corynebacterium* the property of having an unusual cell envelope composition and architecture [7]. The mycolata have a thick peptidoglycan layer, covered by lipids in form of mycolic acids and other lipids [8]–[10]. The mycolic acids are covalently linked to the arabinogalactan, which is in turn attached to the murein of the cell wall [11]. The chain length of these 2-branched, 3-hydroxylated fatty acids varies considerably within the mycolata. Long mycolic acids have been found in Mycobacteria 60–90 carbon atoms), but they are short in Corynebacteria (22–38 carbon atoms) [12]–[16]. This means that the cell wall of the mycolata forms a permeability barrier and probably has the same function as the outer membrane of gram-negative bacteria. These membranes contain channel-forming proteins for the passage of hydrophilic solutes [17]–[19]. Similarly, channels are present in the mycolic acid layer of the mycobacterial cell wall and the cell walls of a variety of Corynebacteria, such as *C. glutamicum*, *Corynebacterium efficiens*, *C. callunae*, and *Corynebacterium diphtheriae* [20]–[25]. In all these cases it seems conceivable that PorA and PorH form heterooligomers responsible for the cation-selective major cell wall channel besides a smaller anion-selective channel [26], [27]. Cell wall channels define the mycolic acid layer as a permeability barrier on the surface of the Corynebacteria similar as has been found in recent years by the investigation of cell wall channels in different members of the mycolata [24], [28]–[32].

The genus *Corynebacterium* contains on the other hand only a few pathogens. The main pathogen is *C. diphtheriae* [33], well known as the cause of diphtheria which is an acute, communicable respiratory disease. Other possible pathogens are only *Corynebacterium urealyticum* and *Corynebacterium jeikeium* [34], [35]. *C. jeikeium* is part of the normal microflora of the human skin. It is a lipid-requiring pathogen that is associated with severe nosocomial infections recognized first in 1970 by Johnson and Kaye [36]. *C. jeikeium* is a strictly aerobic gram-positive rod that causes bioprosthetic valve endocarditis with a high mortality rate (33%) [37], [38]. The bacterium may be multidrug-resistant and needs vancomycin for its treatment. Today the knowledge on the complete genome sequence of *C. jeikeium* K411, a clinical isolate originally recovered from the axilla of a bone marrow transplant patient, provides the basis for an in-depth understanding of the physiology of this medically important bacterium [39]. The chromosome of *C. jeikeium* K411 has a size of 2.46 Mbp and comprises 2104 predicted coding regions, of which 68 most likely represent pseudogenes. The chromosomal architecture of *C. jeikeium* K411 revealed a moderate number of genomic rearrangements when compared to other sequenced corynebacterial genomes [39]. These structural differences of the chromosome have been attributed very recently to the phylogenetic position of *C. jeikeium* within the taxonomic tree of the genus *Corynebacterium* [40]. Annotation of the genomic data revealed that the lipophilic phenotype of *C. jeikeium* is caused by the absence of a gene coding for a fatty acid synthase and linked to pathogenicity, and that events of horizontal gene transfer are responsible for multidrug resistance [39]. The annotated genome sequence can be regarded as starting point for comprehensive post-genomic studies at the transcriptomic and proteomic levels [41], [42], but also for the detailed functional analysis of predicted coding regions, for instance the putative porin gene locus of *C. jeikeium* K411.

In this study, we extended the search for cell wall channels to the *C. jeikeium* strain K411 that is a clinical isolate with a known genome [39]. Using lipid bilayer experiments we could demonstrate that the extracts of whole *C. jeikeium* cells contain a protein that forms wide and water-filled channels similar to the porins found in gram-negative bacteria and in other Corynebacteria [17], [20], [43]. The channel-forming protein, named PorACj, was identified within the accessible genome of *C. jeikeium* K411 [39] by using its homology to PorA of *C. glutamicum*. PorACj was expressed in a PorA/PorH-deficient strain of *C. glutamicum* ATCC13032 [20], [44] and purified to homogeneity. The protein is active as a homooligomer in contrast to PorA/PorH of most Corynebacteria, which form heterooligomeric channels [27]. We present in this study the characterization of the first homooligomeric channel-forming protein of the PorA type of a strain within the genus *Corynebacterium*, which is formed in contrast to other cell wall channels from the mycolata by alpha-helical stretches. A phylogenic tree suggests that PorACj could be the ancestor of all known PorA/H proteins from *Corynebacterium* strains.

## Experimental Procedures

### Bacterial Strains and Growth Conditions

The *Corynebacterium* strains *C. glutamicum* ATCC13032 and *C. jeikeium* K411 (obtained from the National Collection of Type Cultures, NCTC, London, UK) were grown in 1000 ml baffled Erlenmeyer flasks containing 250 ml of brain-heart infusion (BHI) media (Becton) and 250 ml Erlenmeyer flasks containing 25 ml BYT medium [45]. Former cultures were stirred on a rotary shaker at 140 rpm and 30°C, latter at 280 rpm and 37°C. *Escherichia coli* NEB5α (New England Biolabs), used for cloning, was grown under standard conditions in Luria broth (LB). If required agar plates and liquid media were supplemented with 20 and 40 µg/ml chloramphenicol, respectively.

### Cloning of *jk0268*

The gene *jk0268* and its putative ribosome binding site was PCR amplified from genomic *C. jeikeium* DNA. It was isolated in a 50 µl reaction volume containing 1× Taq buffer, 0.2 mM dNTPs, 3 mM MgCl_2_, 1 U Taq DNA polymerase (Fermentas) and 0.4 µM primers FP JK0268XbaI and RP JK0268EcoRI (Table 1). Used PCR conditions were: initial denaturing at 95°C for 5 minutes, 30 cycles at 95°C for 1 min, 45°C for 1 minutes, 72°C for 1 min and a final extension at 72°C for 10 minutes. A PCR product of ∼ 200 bp was cut out after agarose gel size separation, ligated into TOPO2.1 vector (Invitrogen) and heat-shock transformed into *E. coli* NEB5α competent cells according to the instructions of the manufacturers. One plasmid containing the amplification was *Eco*RI and *Xba*I (Fermentas) digested and the 200 bp fragment was ligated in the backbone of *Eco*RI and *Xba*I linearized vector pXMJ19 [46] eventually resulting in the expression plasmid pXJK0268.

**Table 1:** Oligonucleotides used in this study.

Oligonucleotides	Sequence 5′→3′	Position
FP JK0268XbaI	GGAACCTGGCGCTCTAGATCTCTTAAGAGGA	329071–329101
RP JK0268EcoRI	GAAGCCGGGGTTTGAATTCTTAAGCGGAAGC	329232–329262
RP JK0268KpnI	TAAGCGGAGGTACCCTTAGCAGCGGTCCACTTAACG	–
FP KpnIXa8HisEcoRI	CATCGAGGGCCGCGGCGGCCACCACCACCACCACCACCACCACTAATAGG	–
RP KpnIXa8HisEcoRI	AATTC CTATTAGTGGTGGTGGTGGTGGTGGTGGTGGCCGCCGCGGCCCTCGATGGTAC	–
FP pXMJ19Seq	GTGAGCGGATAACAATTTCAC	–
RP pXMJ19Insert	CTCTCATCCGCCAAAACAGC	–
Fwd GST-jk Seq	CAC TCC CGT TCT GGA TAA TG	–
Rev GST-jk Seq	CAC TCC GCT ATC GCT ACG TGA C	–
Fwd R927G	CTGGTTCCGGGTGGATCCC	–
Rev R927G	GGGATCCACCCGGAACCAG	–

The sequences of the primers were derived from the prospective gene *jk0628* of the cell wall channel and its flanking regions taken from the genome of *C. jeikeium* K411 [39]. Primer binding positions in the chromosome of the accessible genome of *C. jeikeium* K411 (reference sequence NC_007164) are provided.

### Construction of a C-terminal His_8_-tag

For immobilized metal ion affinity purification (IMAC) the vector pXMJ19 was upgraded by introduction of a DNA cassette coding a C-terminal factor Xa (I-E-G-R) linked octa-histidine tag. Therefore, single-stranded oligonucletides FP KpnIXa8HisEcoRI and RP KpnIXa8HisEcoRI were first 5′ phosphorylated, then annealed by a temperature gradient step to provide double-stranded DNA with *Kpn*I and *Eco*RI overhangs and finally ligated in *Kpn*I and *Eco*RI cut pXMJ19 vector (T4 DNA ligase, Fermentas). The resulted plasmid was designated pXHis. To apply the C-terminal tag of plasmid pXHis to *jk0268* the native stop codon was mutated by PCR amplification. Using PCR conditions mentioned before, primers FP pXMJ19Seq, RP JK0268KpnI and template pXJK0268 (pXMJ19 equipped with *jk0268*) provided a ∼ 250 bp fragment. This fragment as well as plasmid pXHis were *Xba*I and *Kpn*I digested, over night ligated (16°C) and named pXJK0268His.

### Expression of Recombinant PorACj-His_8_

Transformation of C. glutamicum ΔAH with pXJK0268His was performed by electroporation using a slightly modified standard electro-transformation method [47]. Heterologous expression of the protein was induced by addition of 1 mM IPTG (isopropyl-beta-D-thiogalactopyranoside) to a liquid culture at the mid-exponential growth phase.

### Construction of an N-terminal GST-tag

Besides the C-terminal octa-histidine tag we introduced also an N-terminal GST-tag for the expression of PorACj in *E. coli*. For this a pGEX-2Texpression vector (Amersham Biosciences, GE Healthcare, UK) was used. Due to the N-terminal fusion, the first methionine was removed and an additional transcription terminator was added to the *jk0268* DNA. Next we engineered an IEGR recognition site for FXa and located immediately before *jk0268* gene to cleave the fusion proteins partner. Sequence analysis was done via double strand DNA sequencing into pCR2.1 vector.(Eurofins MWG Operon, Germany) One pCR2.1-jk0268 plasmid containing the amplified modified DNA fragment was EcoRI and BamHI (NEB) digested and the 200 bp fragment was ligated in the backbone of EcoRI and BamHI linearised vector eventually resulting in the expression plasmid pGEX-2TJK0268. Subsequently, site-directed mutagenesis was utilized in pGEX-2Texpression vector prior to usage, to convert R927 of the plasmid to G in the thrombin cleavage sequence (LVPR|GS CTG GTT CCG CGT GGATCC) to avoid any other cleavage site for Factor Xa protease.

The sequence of all expression vectors were verified by sequencing (Seqlab, Göttingen Germany) prior to transformation of the plasmids into competent porin deficient *C. glutamicum* ATCC 13032 ΔAH or porin deficient *BL21 DE3 Omp8 E. coli* strains.

### Isolation of Cell Wall Proteins

Cell wall-associated proteins were isolated by methods described in details previously [28], [30]. A liquid culture of grown cells was centrifuged (6,000 rpm, 15 minutes, 4°C in Heraeus Minifuge RF centrifuge). The cell pellet was washed twice with 10% culture volume (10 mM Tris, pH8) before cell wall proteins were extracted either by shaking the cells in detergent solution or in a 1∶2 (v/v) mixture of the organic solvents chloroform and methanol. For both extraction methods one part cells (0.3 g wet weight bacterial pellet) was resuspended in five parts detergent solution (1.5 ml 1% LDAO (lauryldimethylamine-oxide), 10 mM Tris, pH8) or organic solvent (1.5 ml chloroform/methanol). After 3 hours agitation at RT cells were sedimented in a table top centrifuge (10 minutes, 4°C, 10,000 rpm) and the pellet was discarded. The detergent supernatant was immediately applied to IMAC purification. The chloroform-methanol mixture had first to be precipitated with 9 times the volume of ice-cold diethyl ether (16 h, −20°C) before the obtained pellet was either resolved in detergent solution (1% LDAO, 10 mM Tris, pH8) or in loading buffer for gel electrophoresis [28].

### IMAC Purification

Histidine-tailed *C. jeikeium* protein was purified to homogeneity by utilization of immobilized metal ion affinity chromatography (IMAC). From detergent treated cells 5 ml of the 1% LDAO supernatant were loaded on Ni-NTA spin columns (Qiagen) equilibrated with buffer 1 (20 mM Tris, 50 mM NaCl, 0.4% LDAO, pH8). After ten washing steps using each 650 µl of buffer 2 ( = buffer1 with 10 mM imidazol) bound protein was eluted from the column with 200 µl buffer 3 ( = buffer 1 with 300 mM imidazol).

### Expression of Recombinant GST-jk0268

The plasmid including desired gene was sequenced afterward and transformed into the porin deficient *BL21 DE3 Omp8 E. coli.* Cells were grown at 37°C in LB medium and induced by 1 mM IPTG. The culture media incubated over night at 26°C after induction. Subsequently, cells were harvested by centrifugation at 4,000 ×g for 20 minutes at 4°C and were resupended in PBS phosphate buffered saline (pH 7.4) then lysed with a high-pressure-homogenizer (2×1500 bar). Unbroken cells removed by centrifugation, supernatant used for purification.

### GST-PorACj Fusion Protein Purification

Purification of GST-PorACj was performed using glutathione sepharose 4B medium, (following batch protocol GST Gene Fusion System Handbook, GE Healthcare). After 5 times washing with buffer A (0.5% Genapol, 100 mM Nacl, 50 mM Tris-Hcl, 1 mM CaCl2, 2.5 mM DTT, pH 7.4) to remove non-bound sample components, the purified GST-fusion protein was eluted by addition of buffer A supplemented with 10 mM Glutathione, pH 8. The protein sample was concentrated using amicon ultra 3 kDa [Millipore] to a concentration of 3 mg/ml GST-PorACj determined using OD280. Uses 30 µl of sample highly specific detect only GST fusion protein. Western blotting was carried out using anti-GST antibody. (Data not shown).

### Protease Xa Cleavage of PorACj-His_8_

Subsequent to IMAC purification the sample contained high imidazol concentrations which strongly inhibit protease Xa (Qiagen) activity. Removal of imidazol was performed by dialysing the sample over night against cleavage buffer (20 mM Tris, 50 mM NaCl, 1 mM CaCl_2_, 0.4% LDAO, pH6.5) using a cellulose membrane with a MWCO of 2 kDa (Spectra/Por 6, Carl Roth, Germany). For cleavage of the poly-histidine-tag 4 units protease Xa (Qiagen) were added to the sample (37°C, over night). The enzyme was removed with the factor Xa removal Kit according Qiagen instructions while the cleaved JK0268 (PorACj) protein was separated from uncleaved PorACj-His_8_ by a second passage through a Ni-NTA filter.

### Protease Xa cleavage of PorACj-GST

The purified GST-PorACj contained 10 mM of glutathione. PorACj was dialysed for 36 h at 4°C with constant stirring in glutathione and DTT free buffer (100 mM NaCl, 50 mM Tris, 0.5% Genapol, 1 mM CaCl2, pH 8.0 at room temperature). Then 2 units FXa protease (NEB) was added for 16 h at 22°C to the protein solution. No DTT was added to the dialyses buffer, because of its absorption for CD measurement. The sample was loaded again onto glutathionsepharose 4B medium and the flow through was collected. A concentration of 0.65 mg/ml pure PorACj was determined using OD280.

### Protein Electrophoresis and Immunoblotting

Protein samples were size separated subsequent to a denaturation step (5 minutes, 95°C) with Tris-Tricine 12% or 16.5% polyacrylamide gels [48]. After electrophoresis gels were either stained with Coomassie Brilliant Blue G-250, or by silver stain [49] or electroblotted [50]. In the latter case, proteins were transferred to a 0.1 µm nitrocellulose membrane (Protran, BA79, Whatman). The blotting was performed for 4–5 minutes in a wet tank blot system (Biorad) with Towbin buffer (25 mM Tris, 192 mM glycine, 20% methanol) at 350 mA current. Unspecific binding sites on the membrane were blocked with 5% skimmed milk in TBS-T buffer (20 mM Tris, 0.01 M NaCl, 0.1% Tween, pH7.5) before probing with the first 1∶5000 diluted monoclonal mouse Anti-his antibody (Amersham Biosciences, UK). Subsequent to multiple TBS-T washing steps the second peroxidase-conjugated Anti-Mouse antibodies (DAKO, Denmark) were added at the same dilution. Attending to the manufacturer’s instructions use of the ECL Western blotting detection system (GE Healthcare, UK) resulted in light emission recorded on autoradiography films (Hyperfilm™MP, GE Healthcare, UK). Dot blot immunodetection was carried out identically without prior SDS-PAGE. Exposure times varied between 10 seconds to 5 minutes as required by the sample.

### Test for Susceptibility to Antibiotics

Sterilized Whatman filter disks with 5 mm diameter were used for qualitative tests. Overnight cultures in the suitable medium were diluted 1∶1,000; then 1 ml of each culture containing approximately 10^7^ cells/ml was spread onto BHI agar medium (Difco) containing 1 mM IPTG. Five-microliter portions of the diluted 1∶1,000 stock solutions of the different antibiotics were deposited to each disk. The diameters of the growth inhibition zones were measured after 16 and 24 h. The concentrations of the antibiotics in the growth inhibition zone experiments were: ampicillin, 100 µg/ml; penicillin G, 100 µg/ml; carbenicillin, 100 µg/ml; ceftazidime 100 µg/ml; ertapenem, 20 µg/ml; imipenem, 10 µg/ml; gentamycin, 25 µg/ml; tetracycline 25 µg/ml; Besides the qualitative evaluation of the antibiotic susceptibility also the minimum concentration (MIC) of antimicrobial agents was measured, which inhibits the growth of the different *C. glutamicum* strains used in this study. The stock concentration of the antibiotics in these experiments were the same as above for the growth inhibition zones.

### CD Measurements

CD measurements were performed on a Jasco J-810 circular dichroism spectropolarimeter using 0.1 cm light pathway cells at room temperature while flushing the cuvette chamber with nitrogen gas. Spectra were recorded from 190–260 nm with a resolution of 1 nm and an acquisition time of 200 nm/minutes. Ten scans were taken for each sample, and the average of these scans was smoothed and stored for further analysis. Blanks of the respective protein-free sample were recorded under the same conditions and subtracted from the protein spectrum before further analysis. Analysis of the CD spectra was performed by expressing the spectra as linear combinations of 4 reference spectra (alpha-helix, beta-sheet, beta-turns and random-coil) as described elsewhere by using public domain programs [51].

### Black Lipid Bilayer Membranes

The methods used for the lipid bilayer experiments have been previously described in detail [52]. In the experimental setup two compartments of a Teflon cell filled with electrolyte solution are connected by a small circular hole with an area of about 0.2 mm^2^. The black lipid bilayer membrane was made by painting a 1% (w/v) diphytanoyl phosphatidylcholine (PC)/*n*-decane solution (Avanti Polar Lipids, Alabaster, USA) across the hole. Ag/AgCl electrodes were used connected in series to a voltage source and a home-made current-to-voltage converter for the electrical measurements. The bandwidth of the instrument was set to 100–300 Hz dependent on the magnitude of the output signal. The amplified signal was monitored with a digital oscilloscope and recorded with a strip chart recorder. All salts were obtained from Merck (Germany) or Sigma-Aldrich (Germany) at analytical grade. The aqueous salt solutions were unbuffered and, if not explicit mentioned, had a pH of around 6. The temperature during all experiments was maintained at 20°C. The zero-current membrane potential measurements were performed as it has been described earlier [53] by establishing a 5-fold salt gradient across membranes containing 10–1000 cell wall channels. Zero-current potentials were measured using a high impedance electrometer (Keithley 617).

## Results

### Cell Wall Proteins Effect the Conductance of Lipid Bilayer Membranes

**Figure 1:**
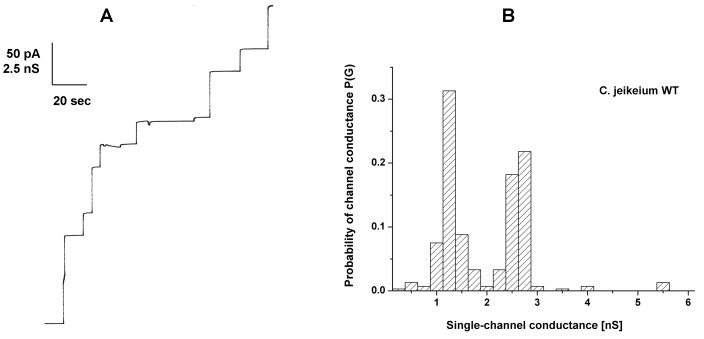
Study of pore-forming capacity of detergent extracts of *C. jeikeium*. (A) Single-channel recording of a PC/*n*-decane membrane in the presence of the detergent extract of whole *C. jeikeium* K411 cells. The aqueous phase contained 1 M KCl, pH 6 and 50 ng/ml protein extract. The applied membrane potential was 20 mV; T = 20°C. (B) Histogram of the probability P(G) for the occurrence of a given conductivity unit observed with membranes formed of 1% PC dissolved in *n*-decane. It was calculated by dividing the number of fluctuations with a given conductance rise by the total number of conductance fluctuations in the presence of detergent extracts of whole *C. jeikeium* K411 cells. Two frequent conductive units were observed for 307 single events taken from 13 individual membranes. The average conductance of the steps corresponding to the left-side maximum was 1.25 nS and that of the right-side maximum was 2.5 nS. The aqueous phase contained 1 M KCl, pH 6 and 50 ng/ml protein extract, the applied membrane potential was 20 mV, T = 20°C.

Cells of an overnight grown *C. jeikeium* culture were extracted with 1% LDAO. A few µl of the crude cell wall extract were tested in the lipid bilayer assay for pore-forming activity (see Figure 1A). Independently, if added to one or both sides of the lipid membranes two discrete conductance steps with 1.25 and 2.5 nS were observed at 20 mV applied membrane potential in 1 M KCl solution (see Figure 1B). Furthermore, the conductance increase caused by the detergent extract was a function of time after the addition of the protein to membranes in the black state. Within about 20 to 30 minutes the membrane conductance increased by several orders of magnitude above that of membranes without the extracts (from about 0.05 µS/cm^2^ to 150 µS/cm^2^). Control experiments with LDAO alone at the same concentration as in the experiments with extracts demonstrated that the membrane activity was caused by the presence of the extracts and not by the detergent. This result suggested that the channel-forming activity was an intrinsic property of the detergent extracts of whole *C. jeikeium* cells.

### Identification of the Gene Coding for the Cell Wall Channel of *C. jeikeium* K411

**Figure 2:**
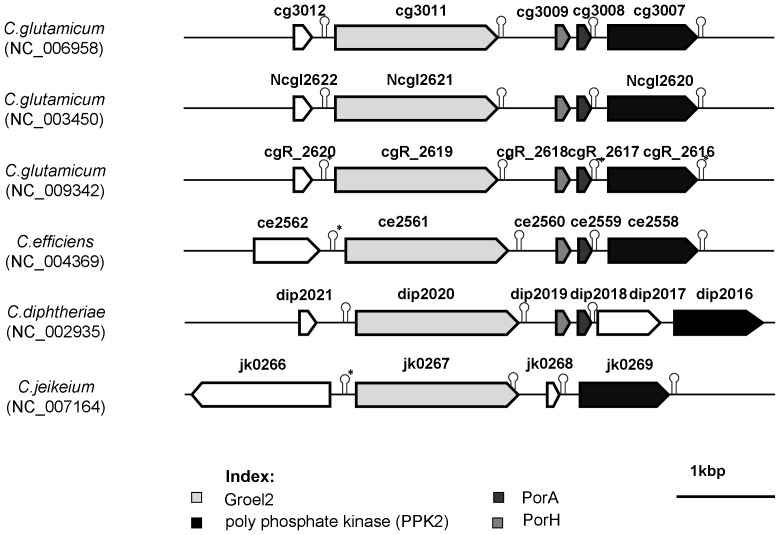
Analysis of the accessible genomes from *C. glutamicum*, *C. efficiens*, *C. diphtheriae* and *C. jeikeium*. The homologous genes of the chaperonin GroEL2 and a poly phosphate kinase PPK2 enclose a presumed conserved porin domain. The operon covering the genes Cg*porH* and Cg*porA* whose proteins build the bicomponental main cell wall channel of *C. glutamicum* is inferred to exist in all strains except for *C. jeikeium*. Possible terminator sequences of mRNA transcripts were predicted with TranstermHP (indicated by hairpins [62]; or were identified manually (marked by asterisk).

The extracts from whole *C. jeikeium* cells contained too many bands in tricine containing SDS-PAGE that it was impossible to relate one single band to the channel-forming activity although it showed a strong band in the low molecular mass region. Excision of the low molecular mass bands from preparative SDS-PAGE suggested that they contained the channel-forming proteins (data not shown). We looked for an alternative method to identify the channel-forming protein. Previously we could identify PorA of *C. diphtheriae* based on its homology with PorA of *C. glutamicum* and other Corynebacteriae [20], [30], [44]. Therefore, we performed a similar approach here. A NCBI BLAST-translation tool search [54], [55] using *porA* of *C. glutamicum* in the known genome of *C. jeikeium* K411 [39] did not lead to a clear indication for a homologous gene. However, search within the genome suggested that it contained an open reading frame (ORF) between the genes coding for GroEL2 (*jk0267*; [56]) and a hypothetical protein (polyphosphate kinase (PKK2), *jk0269*) that could code for a low molecular mass cell wall protein similar to PorA (see Figure 2). This means that an ORF (named *jk0268*) is localized within a region homologous to that of the *C. glutamicum* genome containing *porA* and porH [27]. Primers were designed to clone the whole region between the two genes *jk0267* (GROEL2) and *jk0269* (polyphosphate kinase) using DNA of *C. jeikeium* as a template (see Table 1). The PCR-product was cloned into the TOPO 2.1 vector and was sequenced. It contained *jk0268* that could code for a PorA-like protein.

The involvement of JK0268 (named in the following PorACj for PorA of *C. jeikeium*) in the observed channel formation of *C. jeikeium* extracts was examined by expressing the corresponding gene *jk0268* in a *C. glutamicum* mutant deficient of its main cell wall channel [44]. Whereas in terms of identical treatment cell wall samples of the *C. glutamicum* mutant did not show any channels reconstituted in a planar bilayer membrane, samples of pXJK0268 transformed and IPTG induced mutant cells caused well-defined and step-wise channel events that were very similar to those shown in Figure 1 (data not shown). These channels were identical to those observed after addition of the *C. jeikeium* extracts to lipid bilayer membranes, which means that two maxima of 1.25 and 2.5 nS were observed in 1 M KCl (see Figure 1). This result suggested that PorACj is the cell wall channel of *C. jeikeium*.

### Comparison of PorACj with PorA and PorH of Other Corynebacteriae

**Figure 3:**
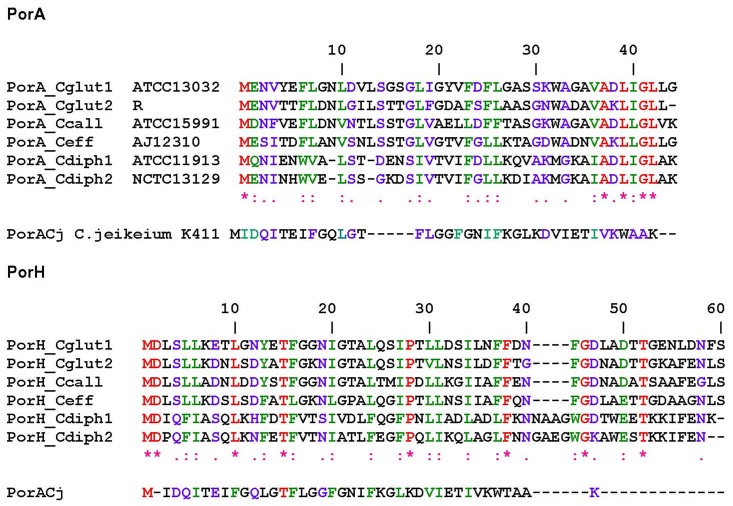
Alignment of PorACj (JK0268) against the class of PorA and PorH proteins of *C. glutamicum* ATCC13032, *C. glutamicum* R, *C. efficiens* ATCC15991, *C. callunae* AJ12310, *C. diphtheriae* ATCC11913 and *C. diphtheriae* NCTC13129. The alignment was performed using Pole Bioinformatique Lyonnaise Network Protein Sequence Analysis (http://npsa-pbil.ibcp.fr) and employing the Clustal W Protein sequences multiple alignments (using the following settings: Gap Opening Penalty: 10; Gap Extension Penalty: 0.2; Protein Weigth Matrix: Gonnet; Gap Separation Distance: 4; Delay Divergent Cutoff (%): 30;). Amino acids identical in all three proteins are highlighted in red (*), strongly similar amino acids (:) are given in green and weakly similar ones (.) in blue. The total number of amino acids is indicated.

Figure 3 shows a comparison of the sequences of the major cell wall proteins PorA and PorH of different Corynebacteriae with that of PorACj derived with Clustal W Protein sequences multiple alignments from Pole Bioinformatique Lyonnaise Network Protein Sequence Analysis (http://npsa-pbil.ibcp.fr). The multiple alignments were controlled with AliBee (www.genebee.msu.su). The protein has similar to PorA and PorH known to date only the inducer methionine but no presequence similar to PorA and PorH of *C. glutamicum* [21], [23], which means that the proteins are exported to the cell wall by a not yet identified mechanism. Similar is also the length of PorACj (40 amino acids) as compared to that of the different PorA (on average 43 amino acids) and PorH (57 amino acids) proteins. Otherwise, the homology is very poor in particular to PorH because only a limited number of amino acids are preserved (see Figure 3). Nevertheless, it is clear that the sequences of the different proteins are related, which means that they are presumably descendents of a common ancestral protein (see Discussion).

### Antibiotic Resistance

The antibiotic susceptibility of *C. glutamicum* ΔHA cells expressing PorACj was examined qualitatively by using the filter disk method and measuring the diameter of growth inhibition zones (see Table 2). The latter method was performed with *C. glutamicum* ATCC13032 wildtype as control, *C. glutamicum* ATCC13032 ΔHA and *C. glutamicum* ΔHA pXHis PorACj. Whereas a considerable decrease of the antibiotic susceptibility was observed between wiltdtype and the ΔHA mutant similar to that observed previously [20], expression of PorACj had an only minor influence on the antibiotic susceptibility of *C. glutamicum* ΔHA pXHis PorACj. Despite expression of the channel former PorACj in *C. glutamicum* ΔHA, the results of antibiotic susceptibility of this mutant strain showed that it was the same for antibiotics such as penicilline G, carbenicillin, ceftazidime, ertapenem and imipenem. Only for ampicillin, gentamicin and tetracycline the *C*. *glutamicum* ΔHA pXHis PorACj strain was slightly more susceptible indicating some minor increase of cell wall permeability in the presence of PorACj.

**Table 2:** Minimum inhibitory concentration (MIC) and diameters of inhibition zones of antimicrobial agents for *C. glutamicum* ΔHA pXjk0268His and *C. glutamicum* ΔHA as control.

Antibiotic	Lowest concentration of antibiotics (MIC) tested that inhibited the growth of bacteria [µg/ml]		Diameters of growth inhibition zones [mm]			
	C. *glutamicum* ΔHA	*C. glutamicum* ΔHA pXjk0268His	*C. glutamicum*ATCC 13032	C. *glutamicum* ΔHA	C. *glutamicum* ΔHApXjk0268his	
Ampicillin	0.39	0.39	21	10	11	
Penicillin G	0.19	0.09	31	23	23	
Carbenicillin	3.1	3.1	11	NI	NI	
Ceftazidime	25	12.5	10	NI	NI	
Ertapenem	0.07	0.07	10	11	11	
Imipenem	2.5	2.5	NI	NI	NI	
Gentamycine	0.15	0.07	16	12	14	
Tetracycline	0.19	0.09	16	12	13	

NI means no inhibition of growth, i.e. no growth inhibition zone.

To determine the minimal concentration of antimicrobial agents, which inhibits the growth of the different *C. glutamicum* strains used in this study, we performed dilution susceptibility tests. This was achieved by dilution of antimicrobial agents in broth media. Antimicrobials were tested in series of two-fold dilutions. The minimum inhibitory concentration (MIC) was measured for *C. glutamicum* ΔHA as a control and *C. glutamicum* ΔHA pXHis PorACj. The results are summarized in Table 2. In general, reduction of antibiotic transport in bacteria using several classes of antibiotics such as β-lactams, aminoglycosides or fluoroquinolones, is mainly caused by changes of membrane permeability [19], [57]. Loss of porins or expression of modified porin structure can cause low uptake of fluoroquinolones and β-lactams. The results of our study demonstrated that *C. glutamicum* ΔHA was still susceptible to all antibiotics. There did also not exist significant differences between *C. glutamicum* ΔHA and *C. glutamicum* ΔHA pXHis PorACj for all antibiotics except for tetracycline, gentamycine and penicillin G. This suggests that *C. glutamicum* ΔHA may have some leakage in the cell wall caused by deletion of its major cell wall channels and therefore did not provide a proper control for the change of cell wall permeability for uptake of antibiotics when PorACj is expressed.

### Heterologous Expression and Purification of the C-terminal His_8_-tagged and the N-terminal GST-tagged Channel-forming Protein PorACj

Cell wall preparations taken either from *C. jeikeium* or *C. glutamicum* expressing PorACj indicated a significant contribution of this protein to the observed channels. For purification of the protein we used a PCR based mutagenesis approach to introduce a DNA sequence coding a protease Xa cleavable, 8 histidine residues comprising protein tail which was added to the C-terminus of the wild-type gene. The constructed DNA cassette, accounting for the *C. glutamicum* codon usage frequencies [58], was introduced in the pXMJ19 expression vector and the stop codon (TAA) of *jk0268* was substituted (GGT) to fuse the peptide tail.

**Figure 4:**
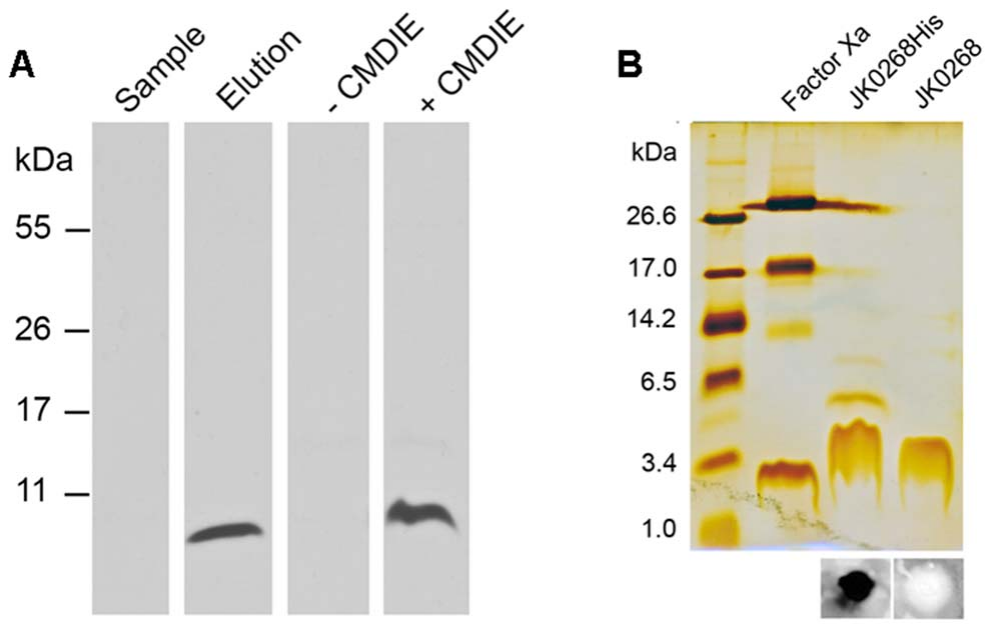
Analysis of PorACj purification. (A) Western blot analysis illustrating IMAC purification of his-tagged PorACj protein. The protein was expressed in *C. glutamicum* ATCC13032 *ΔporHΔporA* and purified by Ni^2+^ affinity from the supernatant of detergent extracted whole cells. CMDIE represents chloroform-methanol treated cells in which the crude protein content was concentrated around 8 fold by diethyl-ether precipitation of pXJK0268His transfected (+) or non-transfected (−) *C. glutamicumΔporHΔporA* cells. Subsequent to tricine (12%)-SDS-PAGE the gel was blotted on a nitrocellulose membrane and PorACj-His was visualized by Anti-His antibodies and a chemiluminescent reaction. All samples were boiled for 5 minutes in Redmix before loading. (B) Silver stained tricine (16.5%)-SDS-PAGE of Ni^2+^-purified and factor Xa digested PorACj-His protein. Lanes: 1, 3 units of protease Xa (control); 2, 10 µl of three pooled Ni-NTA elution containing PorACj-His; 3, 10 µl of protease Xa treated and purified PorACj protein (for details see text). The dot blot immunoassay pictures underneath lanes 2 and 3 show cleavage of the histidine tail using anti-his antibody of 5 µl of the corresponding protein samples. Before loading all samples were boiled for 5 minutes in Redmix.

From IPTG-induced and detergent-extracted *C. glutamicum* cells expressing PorACj it was not possible to directly observe expression of neither the wild-type nor the modified *C. jeikeium* protein in SDS-PAGE. Only higher concentrated samples of chloroform/methanol precipitates combined with immunoassay using an anti-His antibody revealed expression of a small-sized protein as revealed by Western blots with detergent extracts of induced and not induced cells (see Figure 4A). Its molecular mass matched well with the calculated MW of PorACj-His (6.2 kDa). In a next step, the protein was purified from detergent extracts by Ni^2+^ affinity chromatography (see Figure 4B). The pure and still histidine-tagged protein was able to form channels after trace amounts were added to the aqueous phase in the lipid bilayer setup (not studied in detail). To approach the situation of the native protein the His-tag was cleaved by protease Xa and the truncated PorACj protein (containing the C-terminal linker residues G-T-I-E-G-R) was again purified to homogeneity (Figure 4B). Similar results were obtained from lipid bilayer experiments with PorACj as obtained from expression in *E. coli* with the exception that uncleaved GST-PorACj did not form channels (data notshown).

The yield of the expression of the N-terminal GST-PorACj was at least ten times higher than described above for the His_8_-tagged protein. Purification of the GST-tagged PorACj was performed using glutathione Sepharose 4B medium. The protein was eluted with an elution buffer containing 10 mM glutathione. The protein sample was concentrated to a concentration of 3 mg/ml pure GST-PorACj protein as judged by SDS-PAGE and Western blotting. (Data not shown). The GST-tag of the pure protein was removed by cleavage with FXa protease followed by treatment with glutathione Sepharose 4B medium to remove trace amounts of uncleaved GST-PorACj. The final concentration of pure PorACj was 0.65 mg/ml as judged by measurements at OD280. All further biophysical measurements were performed with pure PorACj obtained by cleavage of the His_8_-tag of PorA-His_8_ or by cleavage of the GST-tag of GST-PorACj.

### Single-channel Analysis of PorACj

**Figure 5:**
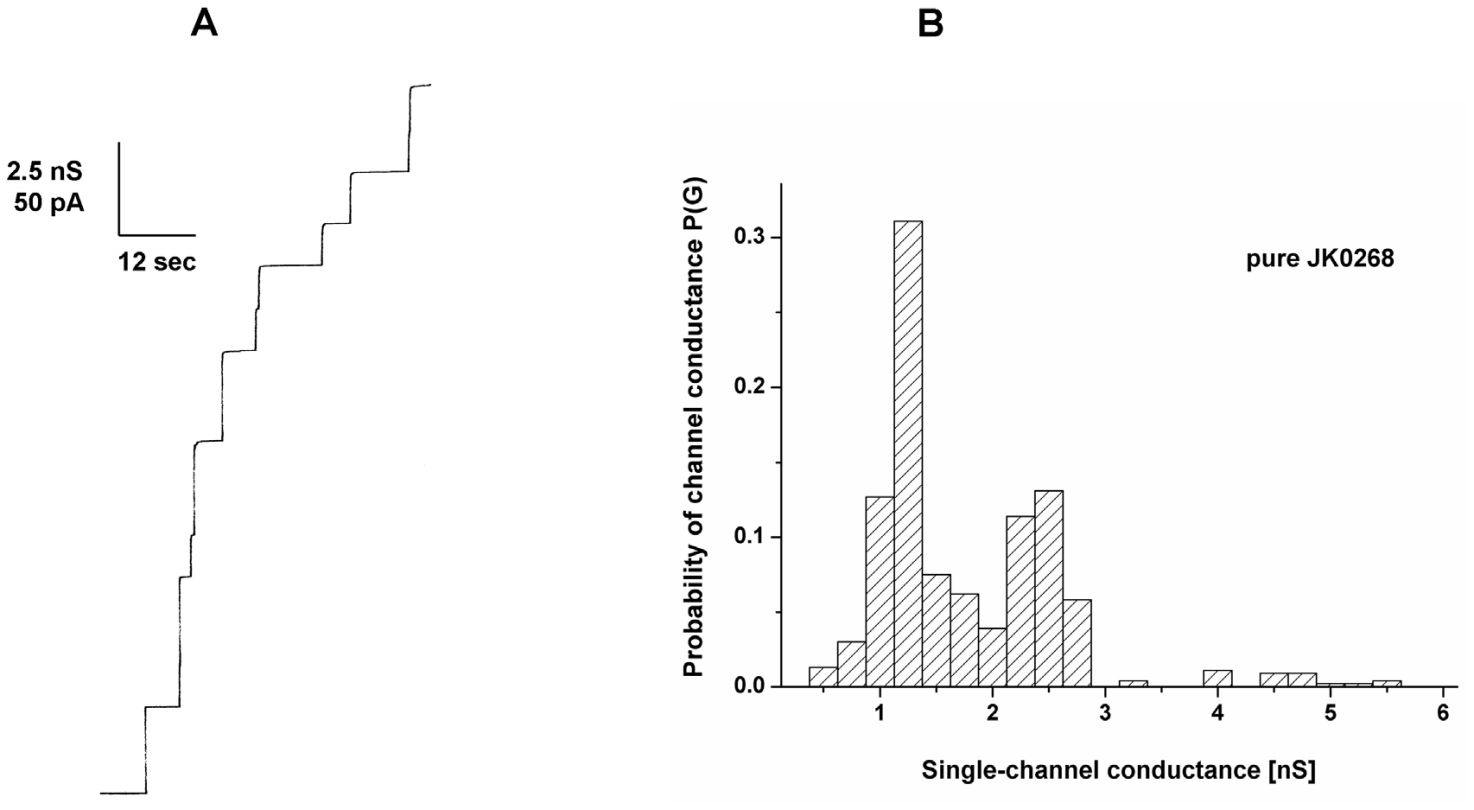
Study of pore-forming capacity of purified PorACj. (A) Single-channel recording of a PC/*n*-decane membrane in the presence of pure PorACj. The aqueous phase contained 1 M KCl, pH 6 and 10 ng/ml protein. The applied membrane potential was 20 mV; T = 20^°^C. (B) Histogram of the probability P(G) for the occurrence of a given conductivity unit observed with membranes formed of 1% PC dissolved in *n*-decane. It was calculated by dividing the number of fluctuations with a given conductance rise by the total number of conductance fluctuations in the presence of pure PorACj. Two frequent conductive units were observed for 295 single events taken from eight individual membranes. The average conductance of the steps corresponding to the left-side maximum was 1.25 nS and that of the right-side maximum was 2.5 nS. The aqueous phase contained 1 M KCl, pH 6 and 10 ng/ml protein, the applied membrane potential was 20 mV, T = 20°C.

Addition of small amounts of the purified PorACj (∼ 10 ng/ml) to one or both sides of a black lipid membrane made of PC/*n*-decane resulted after a few minutes delay in observation of step-like conductance increases. These channels had the same size as described above for detergent-solubilized material from *C. jeikeium* and *C. glutamicum* pXJK0268. This means that most of the steps were directed upwards indicating that the channels were for long time in an open state under low voltage conditions (20 mV; see Figure 5A). Only few channels showed some flickering indicating transitions between open and closed states. The statistical analysis similar to that shown in Figure 1B indicated that most of the channels (more than 40% of all fluctuations) caused conductivity steps with 1.25 or 2.5 nS in 1 M KCl (20 mV applied membrane potential; see Figure 5B). This means that beside a major conductance step of about 1.25 nS we observed also channels with a higher single-channel conductance, in particular channels with a single-channel conductance of about 2.5 nS. It is possible that the two different channels represent two different arrangements of the PorACj monomers (see discussion). Interestingly, we found a 1∶2 relationship for the two maxima within the histograms under all conditions used here, which were denoted as left-side and right-side maximum in the histograms. Single-channel experiments were also performed with salts containing ions other than K^+^ and chloride. These experiments were done to get some insight in the biophysical properties of the cell wall porin of *C. jeikeium*.

The results summarized in Table 3 suggested that the channel may be moderately selective for anions. This conclusion could be derived from experiments in which KCl was replaced by LiCl or KCH_3_COO. The exchange of the mobile ions K^+^ and Cl^-^ by the less mobile ions Li^+^ and acetate^-^ indicates that cations and anions have certain permeability through the channel of *C. jeikeium*, although the effect of anions on the single-channel conductance was more substantial. The permeability of the anions through the channels followed approximately their mobility sequence in the aqueous phase. This probably means that the channels formed by PorACj are wide and water-filled and have only a small field strength inside and no small selectivity filter (i.e. no binding site) as is suggested by the fact that also large organic anions could also penetrate the channel (see Discussion).

**Table 3:** Average single-channel conductance, G, of purified PorACj in different salt solutions.

Salt	Concentration[M]	Single-channel conductance G [pS]	
		left-sidemaximum	right-sidemaximum
KCl	3.0	3,250±230	6,500±410
	1.0	1,250±115	2,500±150
	0.3	350±25	750±55
	0.1	175±15	325±25
	0.03	50±4	120±8
	0.01	33±2	70±5
LiCl	1.0	1,000±85	2,000±140
KCH_3_COO	1.0	500±33	1,100±70
pH 7	0.1	50±6	115±7
KBr	0.1	170±12	340±19
KNO_3_	0.1	140±11	260±16
KClO_3_	0.1	115±9	235±22
KF	0.1	70±5	180±12
KCHOO	0.1	65±4	155±10

The membranes were formed of 1% PC/*n*-decane. The aqueous solutions were unbuffered and had a pH of about 6 if not indicated otherwise. The applied voltage was 20 mV and the temperature 20°C. The single values represent the means (± SD) of at least 100 single-channel events derived from at least four individual membranes.

Table 3 shows also the average single-channel conductance, G, as a function of the KCl concentration in the aqueous phase. The values for G always corresponded to those of the two maxima in the histograms, i.e. to the 1.25 and 2.5 nS peaks in the case of 1 M KCl. Measurements were performed down to 0.01 M KCl. In contrast to other cell wall channels of the mycolata [25], [28], [30], [59], we observed a linear relationship between single-channel conductance and KCl-concentration, which would be expected for wide water-filled channels that do not contain point charges similar to those formed by gram-negative bacterial porins [17], [43]. This means the cell wall channels of *C. jeikeium* are together with those of *C. diphtheriae* [44] the first ones without point charge effects on the channel properties within the taxon Corynebacteriae (see also Discussion).

### Selectivity of the Cell Wall Channel of *C. jeikeium*

Zero-current membrane potential measurements were performed to obtain further information on the structure of the *C. jeikeium* cell wall channel. The experiments were performed in the following way. After the incorporation of 100 to 1000 channels into the PC membranes bathed in 100 mM salt solution, the salt concentration on one side of the membranes was raised fivefold beginning from 100 mM and the zero-current potentials were measured 5 minutes after every increase of the salt gradient across the membrane. For KCl, LiCl and KCH_3_COO the more diluted side of the membrane (100 mM) always became negative, which indicated for all three salts preferential movement of the anions. This result indicates that the channel functions as a general diffusion pore for negative solutes. Analysis of the membrane potential using the Goldman-Hodgkin-Katz equation [53] confirmed the assumption that anions and cations are permeable through the channel. The ratios of the permeability P_cation_ and P_anion_ were 0.34 (KCl), 0.25 (LiCl) and 0.40 (potassium acetate), which means that the selectivity followed the mobility sequence of anions and cations in the aqueous phase, i.e. it is indeed water-filled.

### The Cell Wall Channel of *C. jeikeium* is Voltage-dependent

**Figure 6:**
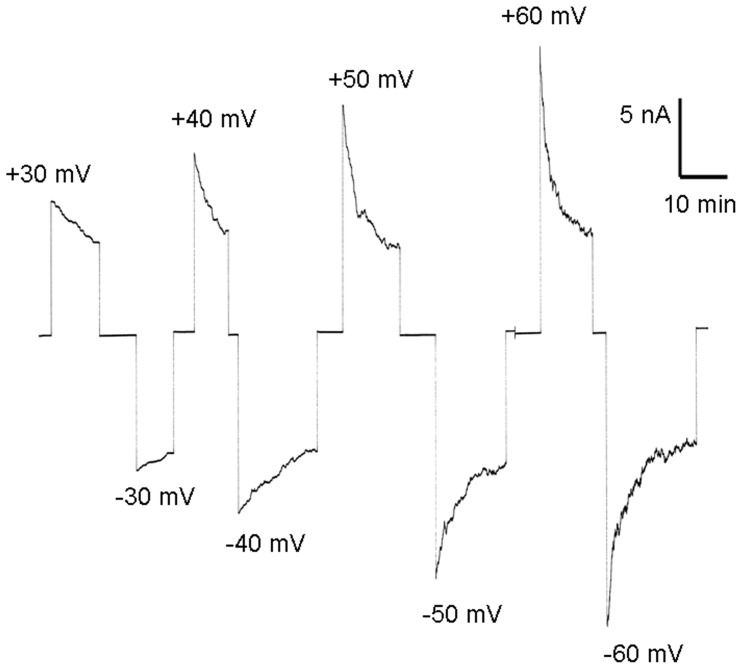
Investigation of the voltage-dependence of PorACj in a multi-channel experiment. The purified protein was added to the *cis*-side of a PC membrane (100 ng/ml) and the reconstitution of channels was followed until equilibrium. Then increasing positive (upper traces) and negative voltages (lower traces) were applied to the *cis*-side of the membrane, and the membrane current was measured as a function of time. The aqueous phase contained 1 M KCl; T = 20°C.

In single-channel recordings, the cell wall porin exhibited some flickering at higher voltages, i.e. it showed rapid transitions between open and closed configurations. This could be caused by voltage-dependent closure of PorACj, which was studied in detail in multi-channel and single-channel experiments. In the first set of experimental conditions, PorACj was added in a concentration of 100 ng/ml to one side of a black PC/*n*-decane membrane (the *cis*-side). After 30 minutes the conductance reached a stationary state. At this time, different positive and negative potentials were applied to the *cis*-side of the membrane. For negative and for positive potentials at the *cis*-side of the membrane the current decreased in an exponential fashion (see Figure 6 for voltages between ±30 mV and ±60 mV). This result indicated symmetrical voltage-dependence of the cell wall channel. The addition of the protein to the *trans*-side of the membrane or to both sides of the membrane also resulted in a symmetric response to the applied voltage (data not shown).

**Figure 7:**
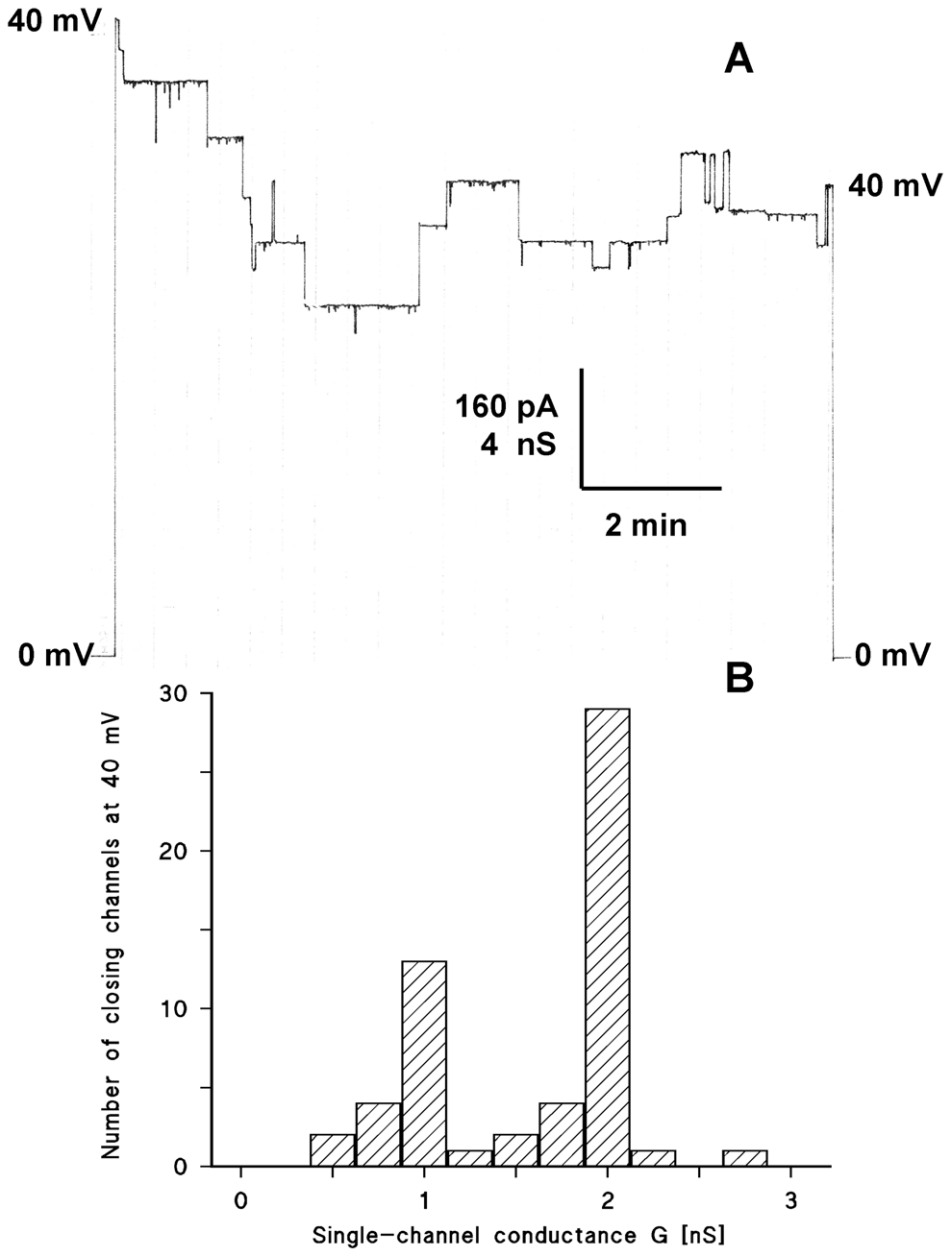
Investigation of the voltage-dependence of PorACj in single-channel experiments. A: The purified protein was added to the *cis*-side of a PC membrane (10 ng/ml) and the reconstitution of channels was followed until about 10 PorACj-channels inserted into the membrane. Then 40 mV were applied to the *cis*-side of the membrane, and the membrane current was measured as a function of time. The aqueous phase contained 1M KCl; T = 20°C. B: Histogram of 56 closing events of the experiment in A and and similar experiments. The closing events were plotted in a bargraph as a function of the conductance of the closing events. ! M KCl; T = 20°C. Note that the PorACj channels closed in two distinct conductance values of 1 and 2 nS.

In a second set of experiments, PorACj was added in a concentration of about 10 ng/ml to one side of a black PC/*n*-decane membrane. After the reconstitution of about 10 channels into the membrane we applied +40 mV to the cis-side of the membrane and followed the time course of the current for about 10 minutes as it is shown in figure 7A. Because of the limited lifetime of the PorACj channel at higher voltages, the channels switched to substates. Figure 7B shows the distribution of the channel closures as a function of the conductance. Two main peaks of conductance were observed. One had a conductance of 1 nS and the other one 2 nS in 1 M KCl. When we keep the onset of conductance with 1.25 and 2.5 nS under the same conditions in mind (see figures 1 and 5), then it is possible that the 1.25 nS channel closes with 1 nS, whereas the 2.5 nS channel closes with 2.0 nS.

**Figure 8:**
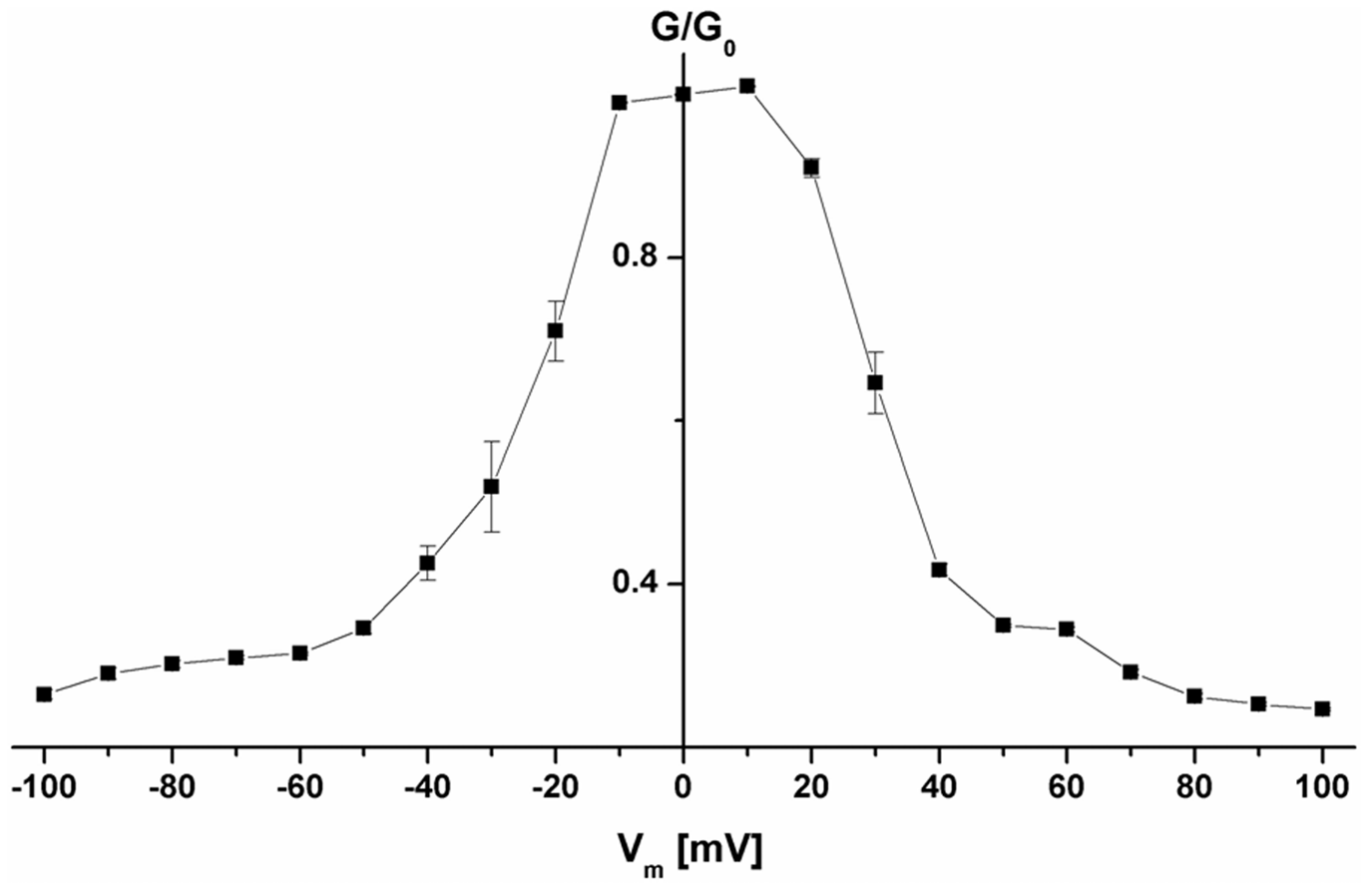
Conductance (G) at a given membrane potential (V_m_) divided by the conductance at 10 **mV (G_0_) expressed as a function of the membrane potential.** The symbols represent the mean (± SD) of six measurements, in which pure PorACj protein was added to the *cis*-side of the membranes. The aqueous phase contained 1 M KCl and 100 ng/ml porin. The membranes were formed from PC/*n*-decane at a temperature of 20°C.

The data of the multi-channel experiments similar to that shown in figure 6 were analyzed in the following way: the membrane conductance (G) as a function of voltage, V_m_, was measured when the opening and closing of channels reached an equilibrium, i.e. after the exponential decay of the membrane current following the voltage step V_m_. G was divided by the initial value of the conductance G_o_, (which was a linear function of the voltage) obtained immediately after the onset of the voltage. The data of figure 8 correspond to the symmetric voltage-dependence of the cell wall porin (mean of four membranes) when the protein was added to the *cis*-side (closed squares). To study the voltage-dependence in more detail the data of Figure 8 were analyzed assuming a Boltzmann distribution between the number of open and closed channels, N_o_ and N_c_, respectively [60]:
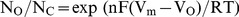
(1)


F, R and T are standard symbols (Faraday constant, gas constant and absolute temperature, respectively), n is the number of charges moving through the entire transmembrane potential gradient for channel gating and V_m_ = V_o_ is the potential at which 50% of the total number of channels are in the closed configuration. The open-to-closed ratio of the channels, N_o_/N_c_, may be calculated from the data in Figure 8 according to
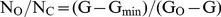
(2)


G is in this equation the conductance at a given membrane potential V_m_, G_o_ and G_min_ are the conductance at 10 mV (conductance of the open state) and at very high potentials, respectively. The data of Figure 8 could be fitted with combination of eqs. (1) and (2). The fit allowed the calculation of the number of gating charges n (number of charges involved in the gating process) and the midpoint potential V_o_ (potential at which the numbers of open and closed channels are identical). The midpoint potential for the addition of the protein to the *cis*-side was for applied positive voltages about +25 mV and for applied negative voltages about −24 mV. The gating charge was in both cases close to 2 (1.9 elementary charges).

### Investigation of the Secondary Structure of PorACj by CD Measurements

**Figure 9:**
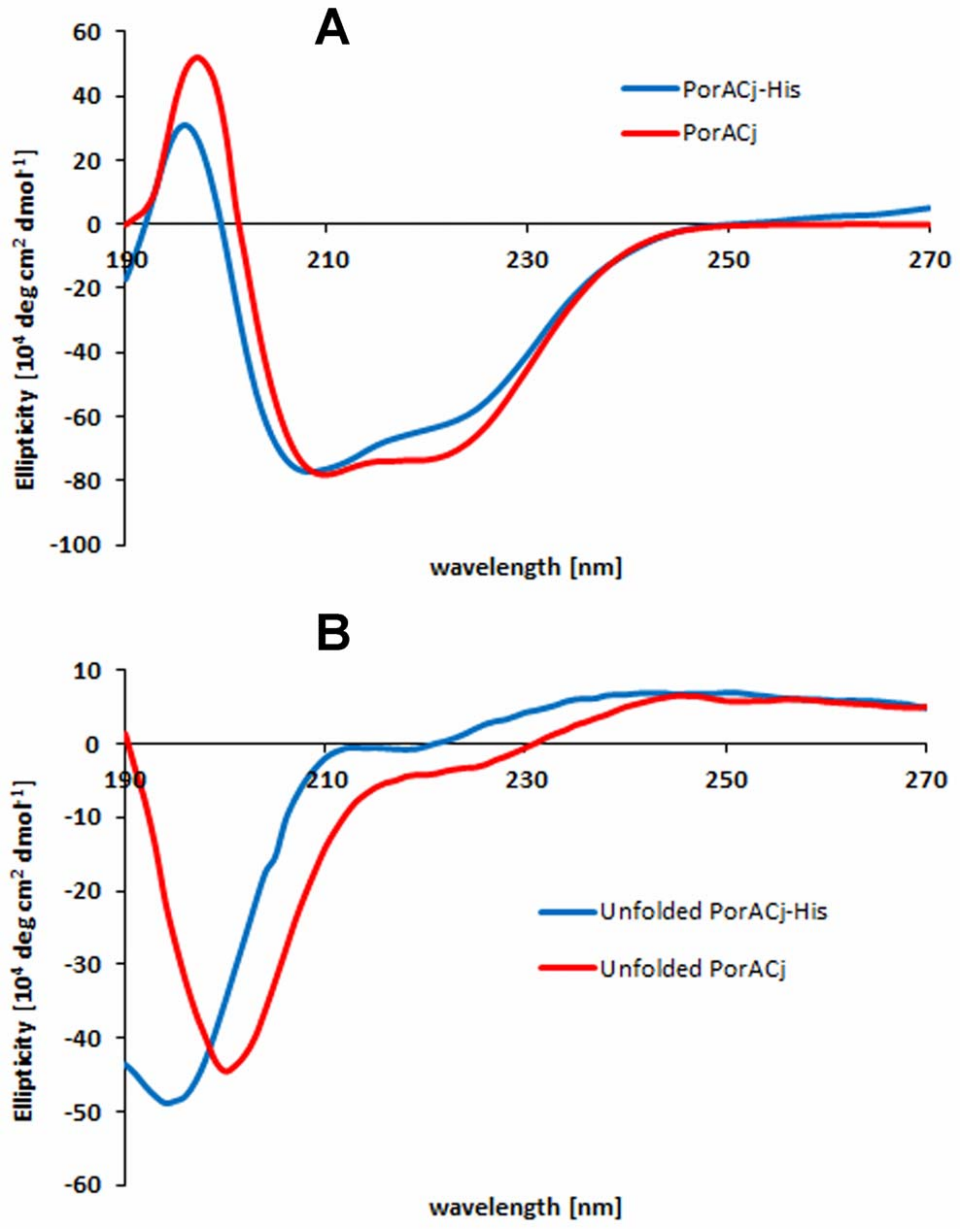
Analysis of secondary structure of PorACj usinf CD-spectrometry. A: CD spectra of recombinant PorACj (69 µM) and PorA-His_8_ (12 µM) solubilized in 0.5% Genapol, 100 mM NaCl, 50 mM TrisHCl and 1 mM CaCl_2_, pH 8 measured at room temperature. B: CD-spectra of the same protein samples as in (A). The aqueous solutions of the proteins was supplemented with 4 M urea to destroy the secondary structure of the proteins.

Secondary structure predictions (http://npsa-pbil.ibcp.fr/cgi-bin/npsa_automat.pl?page=/NPSA/npsa_seccons.html) suggested that the channel formed by PorACj contained alpha-helices in contrast to the known structure of gram-negative bacterial porins [17] and MspA of the gram-positive *Mycobacterium smegmatis* [61]., which are formed by beta-strands Therefore, we considered it as a good model for studies, in which the folding characteristics and the structure of this protein under different conditions were investigated. PorACj and PorACj-His_8_ were solubilized in in a solution containing 100 mM NaCl, 50 mM Tris-HCl and 1 mM CaCl_2_, pH8 supplemented with 0.5% Genapol. The concentrations were 69 µM for PorACj and 12 µM for PorACj-His_8_., which was high enough to obtain a reasonable signal to noise ratio in the CD spectra. For both protein samples we found peaks in the CD-spectra around 208 nm and shoulders at about 220 nm (Figure 9A)., which is typical for a high content of alpha-helical structures in proteins. This was confirmed by the fit procedure of the CD spectra that resulted in an estimate of about 80–100% alpha-helical structure, somewhat dependent on the type of public domain program used for the fit procedure. For control purpose, we performed CD-spectra with the same protein samples at the same concentrations to which urea was added in a concentration of 4 M. Figure 9B demonstrates that the spectra for both PorACj species changed completely. The peaks of the spectra are no between 190 nm and 206 nm indicating that the spectra could be explained by random coil structure.

## Discussion

### The Genome of *C. jeikeium* Contains Only One Gene Coding for a PorA-like Cell Wall Channel

In this work, we extended our study of channel-forming proteins within the family of *Corynebacteriaceae* to the species *C. jeikeium*. Methodologies used previously for the isolation and characterization of cell wall-associated, channel-forming proteins showed that the supernatant of detergent extracted C. *jeikeium* cells also contained channel-forming units that form in the lipid bilayer assay channels with a conductance of 1.25 and 2.5 nS in 1 M KCl (Figure 1). Preparative SDS-PAGE suggested that a small sized protein present in *C. jeikeium* was responsible for the pore-forming activity similar to that of PorH and PorA porins of *C. glutamicum*, *C. callunae*, *C. efficiens* and *C. diphtheriae* [21], [22], [28], [44]. Therefore, the localizations of *porH* and *porA* genes within the chromosomes were compared with the homologous region of *C. jeikeium*. The results suggested that these genes are located in a conserved region flanked by genes coding a chaperonin (GroEL2) and a polyphosphate kinase (PPK2). Applied to *C. jeikeium*, the region between the genes *jk0267* and *jk0269* was astonishingly smaller than that of the other Corynebacteriae. It contained only one open reading frame (ORF), *jk0268* containing 123 nucleotides and coding for a 40AS long polypeptide with a (calculated) MW of 4401 Da. The gene contains 6 bp upstream of the inducer methionine a putative ribosome-binding site (5′-AGGAG-3′). Furthermore, various predicted rho-independent terminator sequences suggested that the gene *jk0268* similar to the situation in the genome of *C. glutamicum* transcribes autonomously of the surrounding genes. Substantiated downstream of *jk0268* by a high-scored (100) stem-loop structure (5′- CCCCGGCTTCGGCCGGGG -3′) upstream structures are probably able to end GroEL2 mRNA transcription (score value 39–66) [62].

It is now clear that the major cell wall channels of most Corynebacteriae, i.e. those of *C. glutamicum*, *C. callunae*, *C. efficiens* and *C. diphtheriae*, are formed by oligomers of two small polypeptides, PorA and PorH [27]. However, this study demonstrates that in contrast to this the major cell wall channel of *C. jeikeium* is formed by an oligomer of a single PorA-like polypeptide. There are two clear findings supporting this. (i) There exists only a single gene (*jk0268*) between *jk0267* (GroEL2) and *jk0269* (PPK2), whereas in the same region within the genomes of the other Corynebacteriae two genes are localized that are transcribed together. (ii) We cloned *jk0268* in expression plasmids. Its expression in a *C. glutamicum* mutant that lacked the genes coding for PorA and PorH resulted in the same channels observed from detergent [or organic solvent] extracts of *C. jeikeium* K411 cells. Similarly, PorACj expressed in *E. coli* resulted in the same channels as the recombinant protein expressed in *C. glutamicum* ΔAH. This result revealed that oligomers of a short 40 amino acids long polypeptide are sufficient to form channels in the cell wall of Corynebacteriae.

Analogous to the situation of PorH and PorA proteins of *C. glutamicum*, *C. callunae*, *C. efficiens* and *C. diphtheriae* the *C. jeikeium* PorACj polypeptide does not contain N- or C-terminal or twin-arginine sorting signals commonly used by gram-positive bacteria for Sec- or TAT-transport [63]–[66]. This means that PorACj uses the same, but still unknown transport mechanism for cell wall proteins of Corynebacteria to overcome the cytoplasmic membrane to reach the cell wall. The single PorACj polypeptide monomers themselves are undoubtedly not large enough to form the observed channels. On the other hand, oligomerization was frequently observed in the field of porin research within mycolate actinomycetes [28], [31], [44], [61].

### PorACj Forms Wide Water-filled Channels with No Indication for Point Charges

We observed two conductance values in the reconstitution experiments with PorACj in all salt solutions (see Table 3). The single channel conductance showed a relation of about 1∶2 and reflected most likely two different channel configurations. A different number of monomers in a PorACj oligomer may cause the two configurations because the pore-forming unit may partially or completely dissociate in detergents or organic solvents during the isolation and purification process. Two configurations of channel closures were also observed in experiments where we studied the voltage dependence of PorACj. However, it is clear from the experiments that the channel did not close completely at higher voltages (see figure 7). This means presumably that the two maxima in the histograms do not reflect the reconstitution of one or two channels at once but reflect two conformers of PorACj.

**Figure 10:**
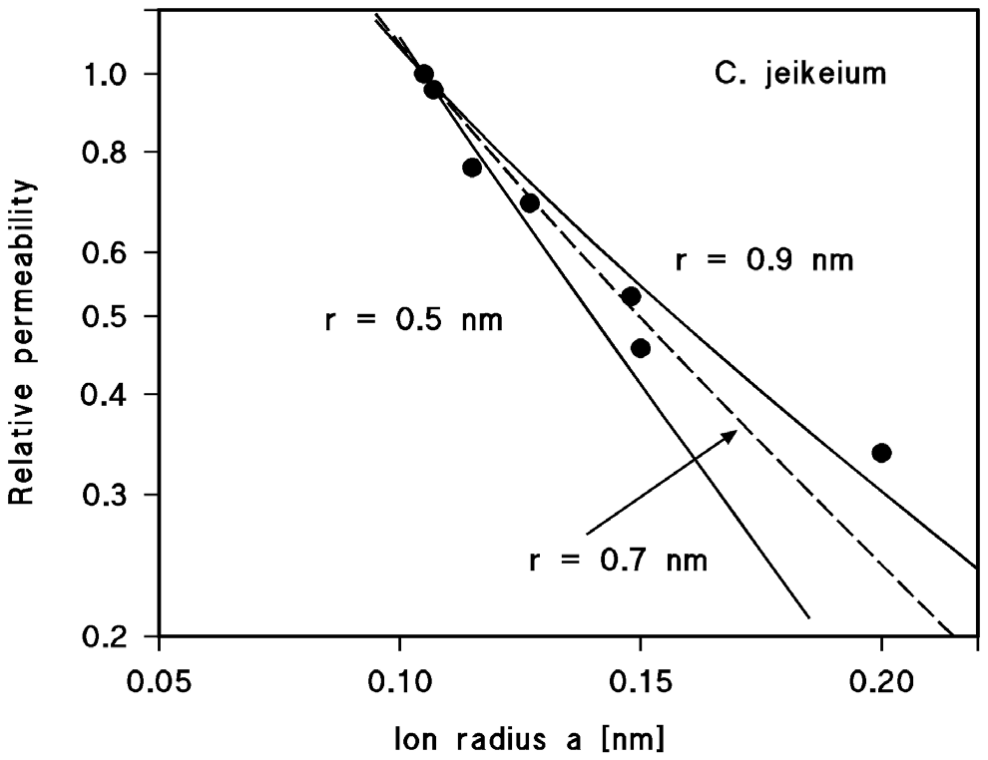
Fit of the single-channel conductance data of PorACj by using the Renkin correction factor times the aqueous diffusion coefficients of the different anions [**67**] **.** The product of both numbers was normalized to 1 for a = 1.05 nm (Br^−^). Single-channel conductance was normalized to the one of 0.1 M KBr and plotted versus the hydrated ion radii taken from Table 3. The single-channel conductance correspond to Br^−^, Cl^−^, NO_3_^−^, ClO_3_^−^, F^−^, HCOO^−^ and CH_3_COO^−^ which were all used for the pore diameter estimation. The fit (solid lines) is shown for r = 0.5 nm (lower line) and r = 0.9 nm (upper line). The best fit was achieved with r = 0.7 nm (diameter = 1.4 nm) corresponding to the broken line.

The conductance of the channels formed by PorACj was a linear function of the bulk aqueous concentration (see Table 3). Similarly, the selectivity of the PorACj channels was dependent on the mobility of the ions in the aqueous phase. This means that the PorACj channels sort mainly according to the molecular mass of the solutes similar to the function of general diffusion pores in gram-negative bacteria [17]. This result is very surprising because up to date many cell wall channels within the taxon mycolata were identified that contained charges in or near the channel opening. The channel presented in this study is the second channel within the Corynebacterineae besides PorA/PorH of *C. diphtheriae* [44] that does not contain point charges. This means also that the single channel analysis does not allow estimation of the channel size on the basis of the existence of point net charges as was performed for cell wall channels of Mycobacteria, Nocardia and Corynebacteriae [25], [28], [31]. On the other hand, a rough estimate of the size of the PorACj channel could be provided from a fit of the single channel data for salts of different anions using the Renkin equation [67]. This was possible because the channel was anion selective under all conditions, which means that the single-channel conductance was mostly limited by the permeability of the anions through PorACj. Furthermore, the permeability of the anions moving through the channel followed approximately their mobility sequences in the aqueous phase (Br^−^>Cl^−^>NO_3_^−^>ClO_3_^−^>F^−^>HCOO^−^>CH_3_COO^−^). This means that the anions passing though the channel interior do not interact much with the channel. Table 4 contains the limiting molar conductivity adopted from ref. [68], the hydrated anion radii calculated according the Stokes equation [67] and the single-channel conductance of the *C. jeikeium* porin which was set to unity relative to the conductance in 0.1 KBr. The fit of the normalized single-channel conductance (mean of the left- and right-hand relative permeability) of PorACj with the Renkin correction factor (eqn. (2) of [67]) times the aqueous diffusion coefficient of the corresponding anion is shown in Figure 10. The best fit of the relative permeabilities was obtained with r = 0.7 nm (diameter 1.4 nm). Thus, the *C. jeikeium* porin is ranking into known channel diameters varying from 1.4 over 2.0 to 3.0 nm of different mycolata, such as *Nocardia farcinica*, *Rhodococcus erythropolis* and *Mycobacterium smegmatis* [30], [31], [61]. However, it was considerably smaller than that of the main PorA/PorH cell wall channel from *C. glutamicum* with 2.2 nm [28].

**Table 4:** Radii of the anions and relative permeability of PorACj from *C. jeikeium* in different salt solutions.

Anion	Radii of the hydratedanions a [nm]	Limited molarconductivity λ_i_ [mS/M]	Permeability relative to 0.1 M KBr	
			left-side maximum	right-side maximum
Br^−^	1.05	78.14	1.0	1.0
Cl^−^	1.07	76.35	1.0	0.96
NO_3_^−^	1.15	71.46	0.82	0.76
ClO_3_^−^	1.27	64.60	0.68	0.69
F^−^	1.48	55.40	0.41	0.53
HCOO^−^	1.50	54.59	0.38	0.46
CH_3_COO^−^	2.00	40.90	0.29	0.34

The data for the limiting conductivities of the different ions were taken from ref. [66]. The radii of the hydrated anions were calculated using the Stokes equation [67]. The single channel conductance of PorACj for the different salts at 0.1 M was taken from Table 3. The relative permeability of the single anions was calculated by dividing the single-channel conductance of the individual anion by that of 0.1 M KBr. The relative permeability for 0.1 M KBr was set to unity.

### Putative Structure of the Channel Formed by PorACj

**Figure 11:**
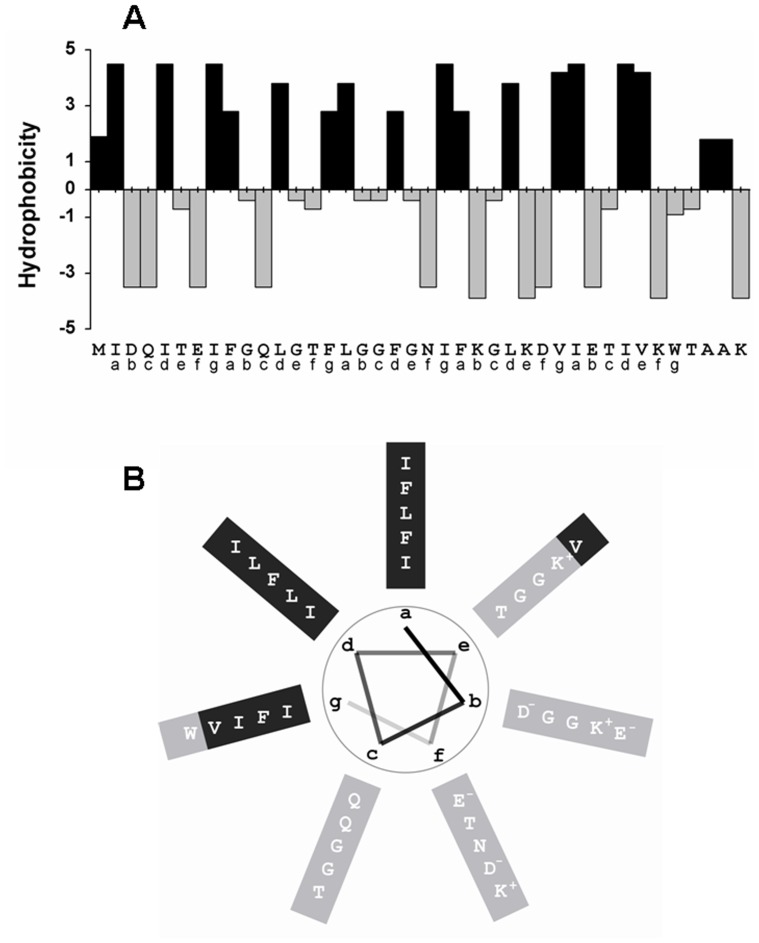
Analysis of PorACj secondary structure. (A) The panel shows the hydrophobicity indices of the individual amino acids of PorACj according to ref [80]. (B) The secondary structure of PorACj was predicted using a consensus method [83] at the Pole Bioinformatique Lyonnaise network (http://npsa-pbil.ibcp.fr/cgi-bin/npsa_automat.pl?page=/NPSA/npsa_seccons.html); the protein was suggested to form α-helices. Amino acid residues arranged on basis of heptameric repeats (a–g) showing distinct separation in a hydrophobic domain supposable surrounded by lipid molecules (dark grey) while the hydrophilic domain (light grey) is suggested to represent the component orientated to the water-filled lumen in the presumed oligomeric PorACj.

**Figure 12:**
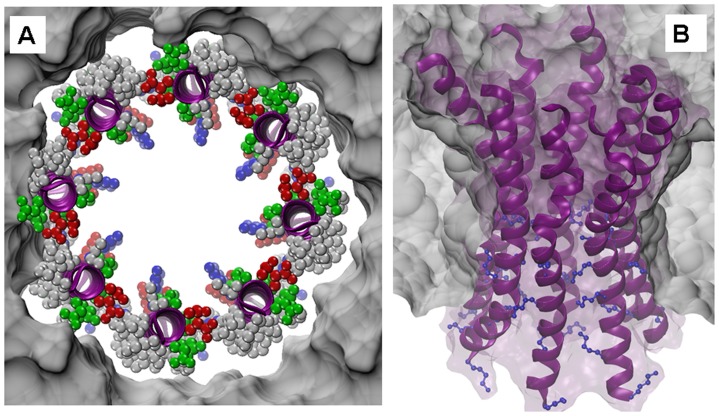
Analysis of the putative quaternary structure of PorACj. (A) Model of the octameric form of the PorACj channel in a lipid bilayer PorACj seen perpendicular to the membrane surface. Top view describes the initial setup of eight straight helices arranged in a circular manner to form a tube with a diameter of about 1.4 nm as derived from the experimental measurements. While the secondary structure is colored in purple, the individual amino-acid side chains are depicted as ball chains and colored according to their electrophysiological nature, i.e., neutral/hydrophobic in grey, polar in green, and charged in red (negative) and blue (positive), respectively. The surrounding bilayer is drawn as a grey surface. (B) Side view of the model of the octameric PorACj channel. After a few tenth of nanoseconds of unbiased molecular dynamics simulations, the helices kink in the central region - forming an hourglass shape - where several short side chains of the amino acids are located. The blue ball-stick side chains represent the lysines in the lower region, which are presumably responsible for the ion selectivity of PorACj and which form some kind of constriction zone.

The comparison of the sequences of different PorA and PorH proteins with PorACj of *C. jeikeium* (Figure 3) demonstrated that the latter one is more comparable in size to the different PorA proteins. In addition, the alignments do not allow a distinctive allocation to PorA or PorH because each class affiliated members show with 13.6% (PorH) and 11.1% (PorA) a remarkable low degree of conserved residues as compared to PorACj. Nevertheless, there existed something like a structural homology between PorACj and the other two channel-forming proteins (PorA/H). Secondary structure predictions of all three proteins suggested that they contain heptameric repeat motive (abcdefg, see Figure 11A) indicating the existence of large α-helical structures with hydrophobic and hydrophilic residues localized on different sides of the helices. Figure 11B shows the possible arrangement of the amino acids in PorACj in an α-helix. This means that this protein could form an amphipathic helix similar to the possible secondary structure in the monomeric PorH and PorA proteins [21], [22], [30]. In agreement with the experimental data (demanding a water filled pore with 1.4 nm diameter), the *C. jeikeium* channel is postulated to consist of oligomeric α-helical subunits. The number of subunits is an open question but this could influence channel conductance. Fig. 12A shows a possible arrangement of PorACj as an octamer seen perpendicular to the axe of the channel. Fig. 12B shows a side view of the octamer. The latter figure was created after 50 ns of unbiased molecular dynamics simulations. A fully stable conformation was not yet achieved at this stage and therefore the present structure suggestion is certainly prelimary (work in progress). Besides an octamer, it is also possible that the channel is formed by a hexamer, which would lead to a considerably smaller single-channel conductance, i.e. two maxima in the channel distribution as we found. We consider the possibility of an uneven number of monomers of PorACj in a channel as rather unlikely because the subunit of the PorA/H channel is presumably a PorA/PorH dimer. The present structure of the channel is definitely in contradiction to the 3D-structure of gram-negative bacterial porins [69] and that of the mycobacterial MspA channel [61] that both form β-barrel cylinders. On the other hand, it represents a similar structure as those of antibiotic channels, such as alamethicin [70], the cell wall porin PorB of *C. glutamicum* [71] and the ligand-gated ion channel in the inner membrane of *Erwinia chrysanthemi* (ELIC) [72].

This could mean that the PorACj monomer is orientated with the leucine, isoleucine and phenylalanine residues (Figure 12B, (g d a)) to the lipid phase, while glycine rich interfaces (c e) allow close contact to neighboring units. The four negative aspartates (D3, D28) and glutamates (E7, E31) together with the four lysines (K24, K27, K35, K40) are oriented to the channel lumen (f b e). At least one of the positive residues must take a dominant position in the otherwise charge-balanced protein causing the determined anion selectivity. The number of PorACj monomers in the homooligomeric channel is not known yet and need further experimental and structural information about the channel. Five monomers are suggested to form the PorB channel of *C. glutamicum* [71] and the ligand-gated channel of prokaryotes [72]. Thus is very likely that the PorACj channel is formed by six to eight monomers because of its larger diameter.

### Is PorACj of *C. jeikeium* the Subunit of the Ancestral Cell Wall Channel of Corynebacteriae?

**Figure 13:**
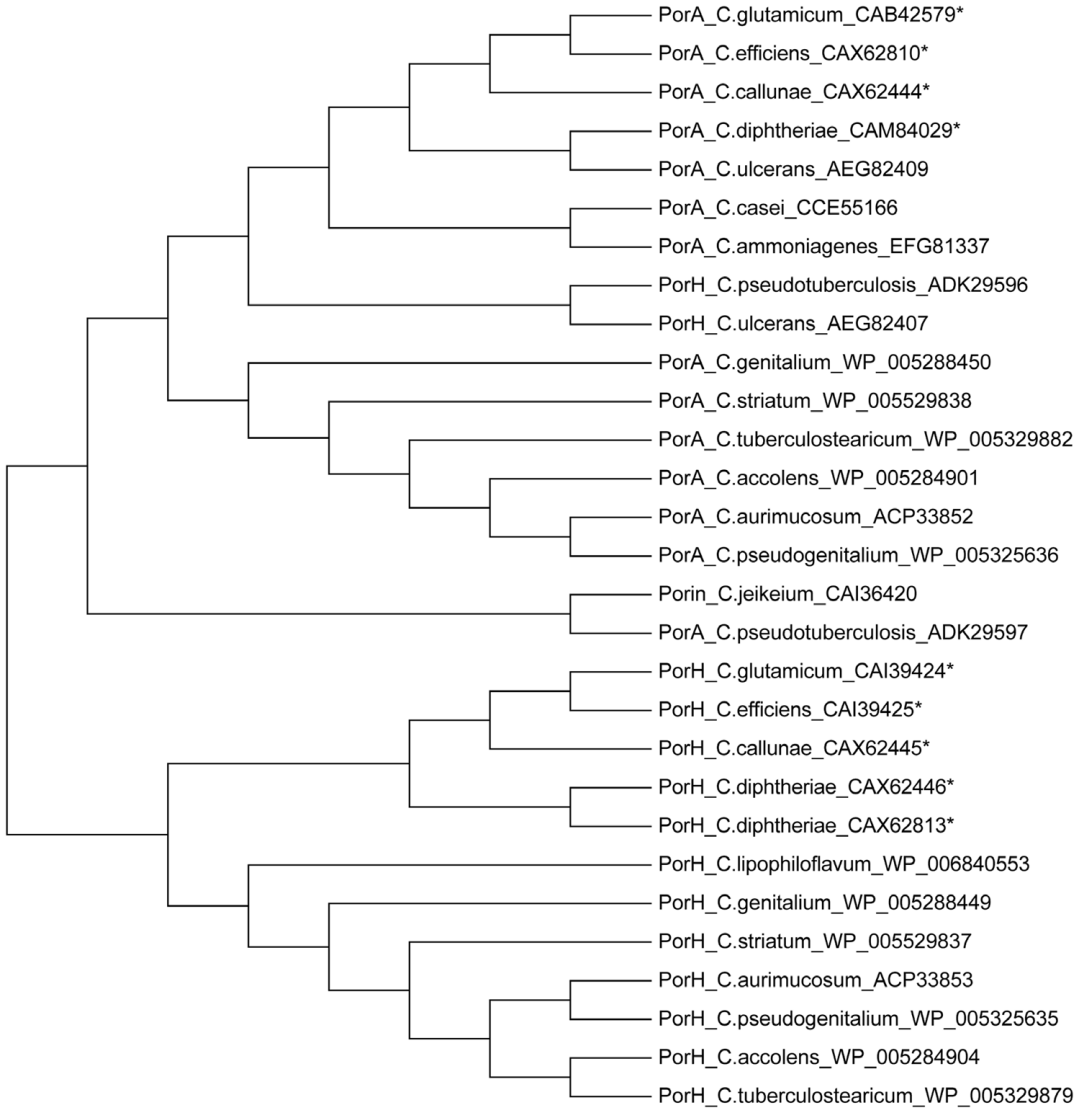
Dendrogram representing the phylogenetic relationships of PorA and PorH of different *Corynebacterium* species obtained by the neighbor-joining method. The tree was derived from the alignments of corresponding gene sequences. The support of each branch, as determined from 1,000 bootstrap samples, is indicated by the value at each node (in percent).The software used to construct alignment and tree was MEGA5.1.The sequence was aligned by ClustalW. Parameters: Multiple Alignment: Gap Opening Penalty: 10; Gap Extension Penalty: 0.2; Protein Weight Matrix: Gonnet; Gap Separation Distance: 4; Delay Divergent Cutoff (%): 30; The phylogenetic tree of corynebacterial species was constructed using the Maximum Likelihood statistical method; Substitution Model; Substitutions Type: Amino acid; Model/Method: Jones-Taylor-Thornton (JTT) model [81].

The many similarities between PorA/PorH and PorACj suggest that these channel-forming proteins form a family of proteins analogous to the MspABCD cell wall channel family of *M. smegmatis* and related species [58], [73]–[75]. Even though PorACj structurally differs from the cell wall channel composition of the heterooligomeric channels within in the genus *Corynebacterium* there is a clear evidence for phylogenic relationships of the investigated species. The different channel characteristics (e.g. diameter and selectivity) may indicate mirror adaptation to the wide-spread habitats of *Corynebacterium* species ranging from soil to skin and tissue of plants, animals as far as to man. In the first run mycolata were mainly classified according to properties of the phenotype and the chemical composition of their cell wall (containing meso-diaminopimelic acid, arabinose and glucose as major sugars [76]. Analysis of 16S rRNA provided deeper insight into separation of species within the monophyletic Corynebacteria-Mycobacteria-Nocardia-group although it cannot claim absolute classification accuracy [77]–[79]. Hence, strains yet known to be concomitant of *porH* and *porA* genes, namely *C. glutamicum*, *C. efficiens*, *C. diphtheriae, C. callunae* and many others likely evolved from an ancestor of *C. jeikeium* [78]. A similar picture may also be derived from a phylogenetic tree of the PorA/H family of channel-forming proteins together with PorACj from the genus *Corynebacterium* shown in Figure 13. The distance between PorACj of *C. jeikeium* and PorA/H of many *Corynebacterium* species is similar. This could indeed mean that *jk0268* (*porACj*) of *C. jeikeium* could be related to the ancestor of the genes *porH* and *porA* that may have evolved by gene duplication. The interesting point in this relationship is that the PorA family of proteins is more closely related to PorACj than the PorH family (see Figure 13).

## References

[1] Eggeling L, Sahm H (1999) L-glutamate and L-lysine: traditional products with impetuous developments. Appl Microbiol Biotechnol 52: 146–153.

[2] Keilhauer C, Eggeling L, Sahm H (1993) Isoleucine synthesis in *Corynebacterium glutamicum*: molecular analysis of the ilvB-ilvN-ilvC operon. J Bacteriol 1757: 5595–603. 10.1128/jb.175.17.5595-5603.1993 PMC206616 8366043

[3] Kinoshita S, Udaka S, Shimono M (1957) Studies on the amino acid fermentation. Part 1. Production of L-glutamic acid by various microorganisms. J Gen Appl Microbiol 50: p. 331–43. 15965888

[4] Leuchtenberger W (1996) Amino acids - technical production and use. Products of primary metabolism, in Biotechnology, Rehm HJ, et al., Editors., Wiley-VCH: Weinheim. p. 465–502.

[5] Sahm H, Eggeling L, Eikmanns B, Krämer R (1996) Construction of L-lysine-, L-threonine-, and L-isoleucine-overproducing strains of *Corynebacterium glutamicum*. Ann N Y Acad Sci. 782: 25–39. 10.1111/j.1749-6632.1996.tb40544.x 8659901

[6] Udaka S (1960) Screening method for microorganisms accumulating metabolites and its use in the isolation of *Micrococcus glutamicus* . J Bacteriol 79: 754–5. 13840150 10.1128/jb.79.5.754-755.1960 PMC278770

[7] Daffe M, Draper P (1998) The envelope layers of mycobacteria with reference to their pathogenicity. Adv Microb Physiol 39: 131–203. 9328647 10.1016/s0065-2911(08)60016-8

[8] Barksdale L (1981) The genus *Corynebacterium*, in The prokaryotes, Starr MP et al., Editors. Springer-Verlag: Berlin. p. 1827–1837.

[9] Goodfellow M, Collins MD, Minnikin DE (1976) Thin-layer chromatographic analysis of mycolic acid and other long-chain components in whole-organism methanolysates of coryneform and related taxa. J Gen Microbiol 96: 351–8. 825611 10.1099/00221287-96-2-351

[10] Ochi K (1995) Phylogenetic analysis of mycolic acid-containing wall-chemotype IV actinomycetes and allied taxa by partial sequencing of ribosomal protein AT-L30. Int J Syst Bacteriol 45: 653–60. 7547284 10.1099/00207713-45-4-653

[11] Minnikin DE (1987) Chemical targets in cell envelops, in Chemotherapy of tropical diseases, Hopper M., Editor., John Wiley & Sons Ltd.: Chichester. p. 19–43.

[12] Brennan PJ, Nikaido H (1995) The envelope of mycobacteria. Annu Rev Biochem 64: 29–63. 7574484 10.1146/annurev.bi.64.070195.000333

[13] Daffe M, Brennan PJ, McNeil M (1990) Predominant structural features of the cell wall arabinogalactan of *Mycobacterium tuberculosis* as revealed through characterization of oligoglycosyl alditol fragments by gas chromatography/mass spectrometry and by 1H and 13C NMR analyses. J Biol Chem 265: 6734–43. 2108960

[14] Holt JG, Kreig NR, Sneath PH, Stanley JT, Williams ST (1994). Nocardioform actinomycetes, in Bergey`s manual of determinative biology, 9th edn. 1994, The Williams and Wilkins Co.: Baltimore. p. 625–650.

[15] Minnikin DE, Patel P, Goodfellow M (1974) Mycolic acids of representative strains of *Nocardia* and the ‘rhodochrous’ complex. FEBS Lett 39: 322–324. 4853302 10.1016/0014-5793(74)80140-7

[16] Yano I, Saito K (1972) Gas chromatographic and mass spectrometric analysis of molecular species of corynomycolic acids from *Corynebacterium ulcerans*. FEBS Lett,. 23(3): p. 352–356. 10.1016/0014-5793(72)80314-4 11946651

[17] Benz R (1994) Solute uptake through the bacterial outer membrane, in Bacterial cell wall, Ghuysen JM, Hakenbeck R Editors., Elsevier Sciene B.V.: Amsterdam. p. 397–423.

[18] Nikaido H (1992) Porins and specific channels of bacterial outer membranes. Mol Microbiol 6: 435–42. 1373213 10.1111/j.1365-2958.1992.tb01487.x

[19] Nikaido H (2003) Molecular basis of bacterial outer membrane permeability revisited. Microbiol Mol Biol Rev 67: 593–656. 14665678 10.1128/MMBR.67.4.593-656.2003 PMC309051

[20] Costa-Riu N, Burkovski A, Krämer R, Benz R (2003) PorA represents the major cell wall channel of the gram-positive bacterium *Corynebacterium glutamicum* . J Bact 185: 4779–4786. 12896997 10.1128/JB.185.16.4779-4786.2003 PMC166458

[21] Hunten P, Costa-Riu N, Palm D, Lottspeich F, Benz R (2005) Identification and characterization of PorH, a new cell wall channel of *Corynebacterium glutamicum*. Biochim Biophys Acta. 1715, 25–36. 10.1016/j.bbamem.2005.07.011 16112217

[22] Hunten P, Schiffler B, Lottspeich F, Benz R (2005) PorH, a new channel-forming protein present in the cell wall of *Corynebacterium efficiens* and *Corynebacterium callunae*. Microbiology. 151: 2429–2438. 10.1099/mic.0.27903-0 16000733

[23] Lichtinger T, Rieß F, Burkovski A, Engelbrecht F, Hesse D, et al (2001) The cell wall channel of the Gram-positive *Corynebacterium glutamicum*: immunological localization and molecular cloning and sequencing of its gene *porA* . Eur J Biochem 268: 462–469. 11168383 10.1046/j.1432-1033.2001.01913.x

[24] Trias J, Jarlier V, Benz R (1992) Porins in the cell wall of mycobacteria. Science 258: 1479–81. 1279810 10.1126/science.1279810

[25] Trias J, Benz R (1993) Characterization of the channel formed by the mycobacterial porin in lipid bilayer membranes. Demonstration of voltage gating and of negative point charges at the channel mouth. J Biol Chem 268: 6234–40. 7681063

[26] Costa-Riu N, Maier E, Burkovski A, Krämer R, Lottspeich F, et al (2003) Identification of an anion-specific channel in the cell wall of the gram-positive bacterium *Corynebacterium glutamicum* . Mol Microbiol 50: 1295–1308. 14622416 10.1046/j.1365-2958.2003.03754.x

[27] Barth E, Barcelo M, Kläckta C, Benz R (2010) Reconstitution experiments and gene deletions reveal the existence of two-component major cell wall channels in the genus corynebacterium. J Bacteriology 192: 786–800. 10.1128/JB.01142-09 PMC2812442 19966008

[28] Lichtinger T, Burkovski A, Niederweis M, Krämer R, Benz R (1998) Biochemical and biophysical characterization of the cell wall channel (porin) of *Corynebacterium glutamicum*: the channel is formed by a low molecular mass subunit. Biochemistry 37, 15024–15032. 10.1021/bi980961e 9790664

[29] Lichtinger T, Heym B, Maier E, Eichner H, Cole ST, et al (1999) Evidence for a small anion-selective channel in the cell wall of *Mycobacterium bovis* BCG besides a wide cation-selective pore. FEBS Lett. 454: 349–355. 10.1016/s0014-5793(99)00844-3 10431837

[30] Lichtinger T, Reiss G, Benz R (2000) Biochemical identification and biophysical characterization of a channel-forming protein from *Rhodococcus erythropolis* . J Bacteriol 182: 764–70. 10633112 10.1128/jb.182.3.764-770.2000 PMC94341

[31] Rieß FG, Lichtinger T, Cseh R, Yassin AF, Schaal KP, et al (1998) The cell wall channel of *Nocardia farcinica*: Biochemical identification of the channel-forming protein and biophysical characterization of the channel properties.Mol Microbiol. 29: 139–150. 10.1046/j.1365-2958.1998.00914.x 9701809

[32] Riess FG, Benz R (2000) Discovery of a novel channel-forming protein in the cell wall of the non-pathogenic *Nocardia corynebacteroides* . Biochim Biophys Acta 1509: 485–95. 11118557 10.1016/s0005-2736(00)00332-1

[33] MacGregor RR (1995) *Corynebacterium diphtheriae*, in Principles and practices of infectious diseases, 4th edn., Mandell GL, Bennett JE, Dolin R, Editors., Churchill Livingston: New York. p. 1865–1872.

[34] Jackman PJ, Pelczynska S (1986) Characterization of *Corynebacterium* group JK by whole-cell protein patterns. J Gen Microbiol 132: 1911–5. 3794641 10.1099/00221287-132-7-1911

[35] Oteo J, Aracil B, Ignacio Alós J, Luis Gómez-Garcés J (2001) [Significant bacteremias by *Corynebacterium amycolatum*: an emergent pathogen]. Enferm Infecc Microbiol Clin 19: 103–6. 11333587 10.1016/s0213-005x(01)72578-5

[36] Johnson WD, Kaye D (1970) Serious infections caused by diphtheroids. Ann N Y Acad Sci, 1970. 174(2): p. 568–76. 10.1111/j.1749-6632.1970.tb45582.x 5278136

[37] Funke G, von Graevenitz A, Clarridge JE 3rd, Bernard KA (1997) Clinical microbiology of coryneform bacteria. Clin Microbiol Rev,. 10(1): p. 125–59. 10.1128/cmr.10.1.125 PMC172946 8993861

[38] Mookadam F, Cikes M, Baddour LM, Tleyjeh IM, Mookadam M (2006) *Corynebacterium jeikeium* endocarditis: a systematic overview spanning four decades. Eur J Clin Microbiol Infect Dis 25: 349–53. 16767481 10.1007/s10096-006-0145-8

[39] Tauch A, Kaiser O, Hain T, Goesmann A, Weisshaar B, et al (2005) Complete genome sequence and analysis of the multiresistant nosocomial pathogen *Corynebacterium jeikeium* K411, a lipid-requiring bacterium of the human skin flora. J Bacteriol 187: 4671–82. 15968079 10.1128/JB.187.13.4671-4682.2005 PMC1151758

[40] Tauch A, Trost E, Tilker A, Ludewig U, Schneiker S, et al (2008) The lifestyle of *Corynebacterium urealyticum* derived from its complete genome sequence established by pyrosequencing. J Biotechnol 136: 11–21. 18367281 10.1016/j.jbiotec.2008.02.009

[41] Brune I, Becker A, Paarmann D, Albersmeier A, Kalinowski J, et al (2006) Under the influence of the active deodorant ingredient 4-hydroxy-3-methoxybenzyl alcohol, the skin bacterium *Corynebacterium jeikeium* moderately responds with differential gene expression. J Biotechnol 127: 21–33. 16890319 10.1016/j.jbiotec.2006.06.011

[42] Hansmeier N, Chao TC, Daschkey S, Müsken M, Kalinowski J, et al (2007) A comprehensive proteome map of the lipid-requiring nosocomial pathogen *Corynebacterium jeikeium* K411. Proteomics 7: 1076–96. 17352426 10.1002/pmic.200600833

[43] Benz R (1988) Structure and function of porins from gram-negative bacteria. Annu Rev Microbiol 42: 359–93. 2849372 10.1146/annurev.mi.42.100188.002043

[44] Schiffler B, Barth E, Daffé M, Benz R (2007) *Corynebacterium diphtheriae*: identification and characterization of a channel-forming protein in the cell wall. J Bacteriol 189: 7709–19. 17720794 10.1128/JB.00864-07 PMC2168714

[45] Tauch A, Bischoff N, Pühler A, Kalinowski J (2004) Comparative genomics identified two conserved DNA modules in a corynebacterial plasmid family present in clinical isolates of the opportunistic human pathogen *Corynebacterium jeikeium* . Plasmid 52: 102–18. 15336488 10.1016/j.plasmid.2004.05.003

[46] Schäfer A, Schwarzer A, Kalinowski J, Pühler A (1994) Small mobilizable multi-purpose cloning vectors derived from the *Escherichia coli* plasmids pK18 and pK19: selection of defined deletions in the chromosome of *Corynebacterium glutamicum* . Gene 145: 69–73. 8045426 10.1016/0378-1119(94)90324-7

[47] van der Rest ME, Lange C, Molenaar D (1999) Heat shock following electroporation induces highly efficient transformation of *Corynebacterium glutamicum* with xenogeneic plasmid DNA. Appl Microbiol Biotechnol 52: 541–5. 10570802 10.1007/s002530051557

[48] Schägger H, von Jagow G (1987) Tricine-sodium dodecyl sulfate-polyacrylamide gel electrophoresis for the separation of proteins in the range from 1 to 100 kDa. Anal Biochem 166: 368–79. 2449095 10.1016/0003-2697(87)90587-2

[49] Blum H, Beier H, Gross HJ (1987) Improved silver staining of plant proteins, RNA and DNA in polyacrylamide gels. Electrophoresis 8: 93–99.

[50] Towbin H, Staehelin T, Gordon J (1979) Electrophoretic transfer of proteins from polyacrylamide gels to nitrocellulose sheets: procedure and some applications. Proc Natl Acad Sci U S A 76: p. 4350–4. 10.1073/pnas.76.9.4350 PMC411572 388439

[51] Greenfield N, Fasman GD (1969) Computed circular dichroism spectra for the evaluation of protein conformation. Biochemistry 8: 4108–4116. 5346390 10.1021/bi00838a031

[52] Benz R, Janko K, Boos W, Läuger P (1978) Formation of large, ion-permeable membrane channels by the matrix protein (porin) of *Escherichia coli* . Biochim Biophys Acta 511: 305–19. 356882 10.1016/0005-2736(78)90269-9

[53] Benz R, Janko K, Läuger P (1979) Ionic selectivity of pores formed by the matrix protein (porin) of *Escherichia coli* . Biochim Biophys Acta 551: 238–47. 369608 10.1016/0005-2736(89)90002-3

[54] Altschul SF, Gish W, Miller W, Myers EW, Lipman DJ (1990) Basic local alignment search tool. J Mol Biol 215: 403–10. 2231712 10.1016/S0022-2836(05)80360-2

[55] Zhang J, Madden TL (1997) PowerBLAST: a new network BLAST application for interactive or automated sequence analysis and annotation. Genome Res 7: 649–56. 9199938 10.1101/gr.7.6.649 PMC310664

[56] Barreiro C, González-Lavado E, Pátek M, Martín JF (2004) Transcriptional analysis of the groES-groEL1, groEL2, and dnaK genes in *Corynebacterium glutamicum*: characterization of heat shock-induced promoters. J Bacteriol 186: 4813–7. 15231814 10.1128/JB.186.14.4813-4817.2004 PMC438587

[57] De E, Basle A, Jaquinod M, Saint N, Mallea M, et al (2001) A new mechanism of antibiotic resistance in Enterobaceriaceae induced by a structural modification of the major porin, Mol. Microbal. 41: 180–198. 10.1046/j.1365-2958.2001.02501.x 11454211

[58] Hallin PF, Ussery DW (2004) CBS Genome Atlas Database: a dynamic storage for bioinformatic results and sequence data. Bioinformatics 20: 3682–6. 15256401 10.1093/bioinformatics/bth423

[59] Riess FG, Dörner U, Schiffler B, Benz R (2001) Study of the properties of a channel-forming protein of the cell wall of the gram-positive bacterium *Mycobacterium phlei* . J Membr Biol 182: 147–57. 11447506 10.1007/s00232-001-0037-x

[60] Ludwig O, De Pinto V, Palmieri F, Benz R (1986) Pore formation by the mitochondrial porin of rat brain in lipid bilayer membranes. Biochim Biophys Acta 860: 268–76. 2427116 10.1016/0005-2736(86)90523-7

[61] Faller M, Niederweis M, Schulz GE (2004) The structure of a mycobacterial outer-membrane channel. Science 303: 1189–1192. 14976314 10.1126/science.1094114

[62] Kingsford CL, Ayanbule K, Salzberg SL (2007) Rapid, accurate, computational discovery of Rho-independent transcription terminators illuminates their relationship to DNA uptake. Genome Biol 8: R22. 17313685 10.1186/gb-2007-8-2-r22 PMC1852404

[63] Freudl R (1992) Protein secretion in gram-positive bacteria. J Biotechnol 23: 231–40. 1368245 10.1016/0168-1656(92)90072-h

[64] Berks BC, Sargent F, Palmer T (2000) The Tat protein export pathway. Mol Microbiol 35: 260–74. 10652088 10.1046/j.1365-2958.2000.01719.x

[65] van Wely KH, Swaving J, Freudl R, Driessen AJ (2001) Translocation of proteins across the cell envelope of Gram-positive bacteria. FEMS Microbiol Rev 25: 437–54. 11524133 10.1111/j.1574-6976.2001.tb00586.x

[66] Ton-That H, Marraffini LA, Schneewind O (2004) Protein sorting to the cell wall envelope of Gram-positive bacteria. Biochim Biophys Acta 1694: 269–78. 15546671 10.1016/j.bbamcr.2004.04.014

[67] Trias J, Benz R (1994) Permeability of the cell wall of *Mycobacterium smegmatis* . Mol Microbiol 14: 283–90. 7830572 10.1111/j.1365-2958.1994.tb01289.x

[68] Castellan GW (1983) The ionic current in aqueous solutions, in Physical Chemistry. Reading, MA: Addison-Wesley, 769–780.

[69] Schulz GE (2004) The structures of general porins, in Bacterial and Eukaryotic Porins, Benz R, Editor, Wiley-VCH: Weinheim. p. 25–40.

[70] Chugh JK, Wallace BA (2001) Peptaibols: models for ion channels. Biochem Soc Trans 29: 565–70. 11498029 10.1042/bst0290565

[71] Ziegler K, Benz R, Schulz GE (2008) A putative alpha-helical porin from *Corynebacterium glutamicum* . J Mol Biol 379: 482–91. 18462756 10.1016/j.jmb.2008.04.017

[72] Hilf RJ, Dutzler R (2008) X-ray structure of a prokaryotic pentameric ligand-gated ion channel. Nature 452: 375–9. 18322461 10.1038/nature06717

[73] Niederweis M, Ehrt S, Heinz C, Klöcker U, Karosi S, et al (1999) Cloning of the mspA gene encoding a porin from *Mycobacterium smegmatis* . Mol Microbiol 33: 933–45. 10476028 10.1046/j.1365-2958.1999.01472.x

[74] Stephan J, Bender J, Wolschendorf F, Hoffmann C, Roth E, et al. The growth rate of *Mycobacterium smegmatis* depends on sufficient porin-mediated influx of nutrients. Mol Microbiol 58: 714–30. 16238622 10.1111/j.1365-2958.2005.04878.x

[75] Dörner U, Maier E, Benz R (2004) Identification of a cation-specific channel (TipA) in the cell wall of the gram-positive mycolata *Tsukamurella inchonensis*: the gene of the channel-forming protein is identical to mspA of *Mycobacterium smegmatis* and mppA of *Mycobacterium phlei* . Biochim Biophys Acta 1667: 47–55. 15533305 10.1016/j.bbamem.2004.09.001

[76] Lechevalier HA, Lechevalier MP, Gerber NN (1971) Chemical composition as a criterion in the classification of actinomycetes. Adv Appl Microbiol 14: 47–72. 5160678 10.1016/s0065-2164(08)70539-2

[77] Ruimy R, Riegel P, Boiron P, Monteil H, Christen R (1995) Phylogeny of the genus *Corynebacterium* deduced from analyses of small-subunit ribosomal DNA sequences. Int J Syst Bacteriol 45: 740–746. 7547293 10.1099/00207713-45-4-740

[78] Pascual C, Lawson PA, Farrow JA, Gimenez MN, Collins MD (1995) Phylogenetic analysis of the genus *Corynebacterium* based on 16S rRNA gene sequences. Int J Syst Bacteriol 45: 724–728. 7547291 10.1099/00207713-45-4-724

[79] Khamis A, Raoult D, La Scola B (2004) rpoB gene sequencing for identification of *Corynebacterium* species. J Clin Microbiol 42: 3925–31. 15364970 10.1128/JCM.42.9.3925-3931.2004 PMC516356

[80] Kyte J, Doolittle RF (1982) A simple method for displaying the hydropathic character of a protein. J Mol Biol 157: 105–32. 7108955 10.1016/0022-2836(82)90515-0

[81] Tamura K, Peterson D, Peterson N, Stecher G, Nei M, et al (2011) MEGA5: molecular evolutionary genetics analysis using maximum likelihood, evolutionary distance, and maximum parsimony methods. Mol Biol Evol. 28: 2731–2739. 10.1093/molbev/msr121 PMC3203626 21546353

